# Origin, distribution, and potential risk factors associated with influenza A virus in swine in two production systems in Guatemala

**DOI:** 10.1111/irv.12437

**Published:** 2017-01-30

**Authors:** Ana S. Gonzalez‐Reiche, Ana L. Ramírez, María L. Müller, David Orellana, Silvia M. Sosa, Pablo Ola, Jorge Paniagua, Lucía Ortíz, Jorge Hernandez, Celia Cordón‐Rosales, Daniel R. Perez

**Affiliations:** ^1^ Department of Population HealthPoultry Diagnostic and Research Center AthensUniversity of GeorgiaAthensGAUSA; ^2^ Universidad del Valle de GuatemalaGuatemala CityGuatemala; ^3^ Prince Leopold Institute of Tropical MedicineAntwerpBelgium; ^4^ Ministerio de Agricultura Ganadería y AlimentaciónGuatemala cityGuatemala; ^5^ University of FloridaGainesvilleFLUSA; ^6^ Present address: Department of Genetics and Genomic SciencesIcahn School of Medicine at Mount SinaiNew YorkNYUSA; ^7^ Present address: James Cook UniversityTownsville CityQLDAustralia

**Keywords:** Central America, epidemiology, farm, Guatemala, influenza A virus, phylogeny, spatial analysis, swine

## Abstract

**Background:**

Guatemala is the country with the largest swine production in Central America; however, evidence of influenza A virus (IAV) in pigs has not been clearly delineated.

**Objectives:**

In this study, we analyzed the presence and spatial distribution of IAV in commercial and backyard swine populations.

**Methods:**

Samples from two nationwide surveys conducted in 2010 and 2011 were tested using virological (rRT‐PCR and virus isolation) and serological (ELISA and hemagglutination inhibition) assays to detect IAV.

**Results:**

Influenza A virus was detected in 15.7% of the sampled pigs (30.6% of herds) in 2010 and in 11.7% (24.2% of herds) in 2011. The percentage of seropositive pigs was 10.6% (16.1% of herds) and 1.4% (3.1% of herds) for each year, respectively. Three pandemic H1N1 and one seasonal human‐like H3N2 viruses were isolated. Antibodies against viruses from different genetic clusters were detected. No reassortant strains with swine viruses were detected. The H3N2 virus was closely related to human viruses that circulated in Central America in 2010, distinct to the most recent human seasonal vaccine lineages. Spatial clusters of rRT‐PCR positive herds were detected each year by scan statistics.

**Conclusions:**

Our results demonstrate circulation of IAV throughout Guatemala and identify commercial farms, animal health status, and age as potential risk factors associated with IAV infection and exposure. Detection of human‐origin viruses in pigs suggests a role for humans in the molecular epidemiology of IAV in swine in Guatemala and evidences gaps in local animal and human surveillance.

## 1 Introduction

Interspecies transmission events of IAVs between humans and pigs play a significant role in the generation of novel reassortant strains that may spread among humans and/or swine populations.^1^, ^2^ The role of humans in the epidemiology of swine IAV has been increasingly recognized due to the accumulating evidence of reverse zoonotic transmission events observed over the past century.^3^ Information on the prevalence and distribution of swine IAVs remains limited in many regions of the world, particularly in Latin America.^4^ Recent studies suggest that introduction of human viruses into pigs may be a major driver in the evolution of IAV lineages that are exclusive to Latin America.^5^ In general, animal husbandry practices in many of these countries resemble those from other regions that are believed to be associated with an increased risk of exposure to zoonotic influenza viruses.^6^ In Central America, Guatemala is the country with the largest swine industry (estimated population size >2.7 million).^7^ Although large‐scale commercial farms with enclosed housing exist, most swine production is peridomestic—in household backyards or open smallholdings—without specialized equipment. In these systems, pigs are often free ranged or kept in contact with other domestic animals.^8^ Serological evidence of H1N1 and H3N2 in pigs was documented previously^9^; however, only a limited number of strains were used which may have resulted in limited detection of antigenic diversity. In Guatemala, vaccination against swine IAVs is generally not practiced, and it is neither recommended nor regulated by animal health authorities. To our knowledge, virus isolation from animal samples has not been attempted in Guatemala; consequently, the genetic diversity and the distribution of circulating virus strains in the country remain unknown. In this study, two nationwide surveys were performed in pigs in Guatemala to detect IAV. Viral infection and serological exposure were investigated using molecular and serological testing. The viruses’ origin in the sampled population was identified through phylogenetic analysis. Information available on the type of production system, geographic location, and animal characteristics was used to identify potential risk factors and to analyze the spatial distribution of IAV‐positive herds.

## 2 Materials and methods

### 2.1 Sample collection

Two nationwide surveys were conducted in Guatemala, one in 2010 (October) and one in 2011 (June–August). To demonstrate the presence of IAV in the pig population in Guatemala, 500 samples per year were collected throughout the country, sufficient to detect 1% circulation of virus with 99% confidence.^10^ The samples were distributed proportionally to the swine population, by department (administrative subdivisions in Guatemala) and type of pig production system: small‐ to medium‐scale commercial farms, or backyards. Pig production units (PPUs) were recruited under voluntary participation from those registered in the Guatemalan Association of Swine Producers attended by veterinarians from the union of swine technical specialists. Field veterinarian epidemiologists from the Guatemala Ministry of Agriculture, Livestock and Food (MAGA) conducted recruitment, data collection, and sampling. Sampling of animals was conducted under approved animal use protocols from MAGA, and the protocols were reviewed and approved by the Institutional Animal Use and Care Committee of University del Valle de Guatemala. In 2010, samples were collected preferentially from animals showing clinical signs of respiratory disease (nasal/ocular discharge or sneezing). In 2011, sample distribution by geographic location and type of PPU was similar, but sampling from sick animals was not prioritized. For virus detection, nasal swabs were collected and placed in 3 mL of viral transport medium with antibiotics and antimycotics.^11^ Additionally, 2 mL of blood was collected from the orbital sinuous vein for antibody detection. Information on potential risk factors at the animal level (animal health status, age, and sex—only done in 2011) and at the herd level (herd size, type of PPU, geographic location) was collected at the time of sampling.

### 2.2 Virus and antibody detection

Viral RNA was extracted from nasal swab supernatants with the MagMAX‐96 AI/ND Viral RNA Isolation Kit (Ambion, Austin, TX, USA) according to the manufacturer's instructions. Influenza A virus RNA was detected by rRT‐PCR with matrix‐specific primers,^12^, ^13^ using the one‐step RT‐PCR or the Quantitec QuantiTect Probe RT‐PCR Kits (QIAGEN, Hilden, Germany) in the ABI 7300 Real‐Time PCR System (Applied Biosystems, Foster City, CA). Positive controls for matrix RNA detection included RNA extracts from inactivated virus (provided by the National Veterinary Services Laboratories, USDA, Ames, Iowa) or *in vitro* transcribed RNA from plasmid DNA containing the corresponding gene segment. All rRT‐PCR‐positive samples were tested for virus isolation in MDCK cells and 9‐ to 10‐day‐old embryonated chicken eggs. Up to three blind passages were carried out to test for viable virus. Virus isolates were identified by direct full‐length sequencing of all gene segments with influenza A universal primers and compared with BLAST^14^ as previously described.^15^

Serum samples were tested for antibodies against IAV with the commercially available kit, IDEXX ELISA Influenza A Ab (IDEXX, Westbrook, ME). The cutoff value was validated and adjusted.^16^ ELISA‐positive samples were tested by hemagglutination inhibition (HI) assay with standard protocols,^11^ against selected swine and human H1 and H3 viruses from different genetic clusters. Samples were considered positive to the antigen with the highest inhibition titer, and exposure to multiple viruses was considered positive when the inhibition titer was the same for more than one reference antigen and when positive to multiple subtypes.

### 2.3 Statistical analysis

The percentages of positive and seropositive pigs detected by RT‐PCR and ELISA were computed by year, type of PPU, and other collected variables. At the animal level, potential risk factors were tested by generalized estimating equations (GEE), to account for clustered observations from the same herd. An exchangeable correlation structure was assumed using robust variance estimates.^17^ A bivariate model was considered using each risk factor (animal health status, age, or sex) as independent variable and virus detection (rRT‐PCR) or exposure (ELISA) as the dependent variable. Due to demographical differences between the sampled populations, these associations were computed independently for each year and combined when similar results were obtained (adjusting by year).

For all calculations at the herd level, a herd was considered positive when at least one animal tested positive by rRT‐PCR or ELISA. The percentages of positive and seropositive herds were computed by year and type of PPU. To analyze the risk of IAV infection or exposure of a herd, odds ratios (ORs) were estimated by logistic regression. A bivariate analysis was done using type of PPU (farm *vs*. backyard) as independent variable, and virus detection (rRT‐PCR) or exposure (ELISA) as dependent variable. All analyses were performed using the packages stats and geepack v.1.2‐0 in the programming language R version 3.2.2 for Mac OS X.^18^, ^19^

### 2.4 Spatial analysis

For spatial analysis, it was assumed that sampled PPUs are a representative spatial sample of the distribution of swineherds in Guatemala. The global positioning system (GPS) coordinates were validated in Google Earth. When latitude information and longitude information were unavailable, the coordinates were assigned to match the location name according to the National Geographic Institute of Guatemala (http://www.ign.gob.gt). After validation, 34% of the GPS coordinates were corrected in 2010 and 42% in 2011. The spatial scan statistic was used to identify spatial clusters. Space–time analysis was performed using the information of herd location and status (rRT‐PCR positive or negative) from the 2 years. The analyses were performed in SaTScan ^®^ version 9.1.1.1 for Mac OS X.^20^ Areas with high positivity rates were scanned, using a Bernoulli distribution as the probability model. An elliptical window shape was used with a maximum spatial cluster size of 50% of the population at risk and 999 Monte Carlo randomizations.^21^ A robust standard error was used to account for the corrections made in the geographic coordinates, and clusters were considered significant when *P*<.1. Sensitivity analysis was performed using a circular window shape and different maximum scanning window sizes to test for robustness of the clusters found. The clusters were mapped in Manifold 8^®^ system.

### 2.5 Virus sequence characterization and phylogenetic analysis

The Sequence Feature Variant Type tool of the Influenza Research Database (IRD)^22^ was used to search for the presence of markers associated with increased replication and pathogenicity in mammalian hosts in the virus isolates. The viruses were further analyzed for the presence of mutations in comparison with other viruses circulating in the region (Mexico, Central America and the Caribbean) between 2009 and 2011. Protein alignments of the HA1 were performed to identify mutations in the antigenic sites of the isolated viruses.

Phylogenetic analysis was performed for the surface genes (HA and NA). Nucleotide sequences of human H3N2 (from 2007 to 2013) and pandemic H1N1 viruses (from 2009 to 2013) from Central America and the Caribbean were downloaded from the EpiFlu database of the Global Initiative on Sharing All Influenza Data (the search included sequences also available in GenBank, in addition to those only available in EpiFlu) (GISAID, http://platform.gisaid.org, Table S1). Phylogenetic analyses were performed in MEGA 6.0.^23^ Sequences were manually trimmed and final coding sequences were aligned with MUSCLE for codons. After alignment, datasets were subsampled to remove identical sequences and reduce sampling bias. Final phylogenetic trees were constructed using maximum‐likelihood (ML) inference with the best‐fit model of nucleotide substitution determined by the BIC criterion and Hasegawa‐Kishino‐Yano (HKY) with gamma distribution. Robustness of tree topologies was assessed with 100 neighbor‐joining bootstrap replicates.

## 3 Results

### 3.1 Sample collection

Samples were collected from 188 herds in 2010 (500 pigs) and 199 herds in 2011 (499 pigs) (Table 1). Summary statistics of the herd sizes and type of sampled PPUs are shown in Table S2. In 2010, sampled PPUs included commercial farms (45%, n=85, 329 pigs) and backyards (54%, n=101, 169 pigs). In addition, two samples were submitted from one agricultural school and one PPU not identified as farm or backyard. In 2011, samples were submitted from 53 commercial farms (27%, 230 pigs), 141 backyards (71%, 257 pigs), one abattoir (4 pigs), an agricultural school (2 pigs), and two unidentified PPUs (5 and 1 pigs, respectively). Samples that were not from farms or backyards were tested in the laboratory, but they were excluded from all statistical analyses. At the time of sampling in 2010, 61% (n=306) of sampled pigs were reported with respiratory signs, and in 2011 only 22.1% (n=108). Information on animal sex and age was collected only in 2011 (Table 1).

**Table 1:** Characteristics of pigs sampled for IAV in Guatemala, 2010‐2011

Variable	Year				Total	
	2010		2011			
	n^a^	(%)	n	(%)	n	(%)
Sex						
Female	N/A	‐	189	(39)	189	(19)
Male	N/A	‐	254	(52)	254	(26)
Unknown	498	(100)	44	(9)	542	(55)
Age						
Pup	N/A	‐	88	(18)	88	(9)
Weanling	N/A	‐	153	(31)	153	(16)
Juvenile	N/A	‐	45	(9)	45	(5)
Adult	N/A	‐	171	(35)	171	(17)
Unknown	498	(100)	30	(6)	528	(54)
Type of PPU/herd						
Farm	329	(66)	230	(47)	559	(57)
Backyard	169	(34)	257	(53)	426	(43)
Health Status						
Sick	306	(61)	108	(22)	414	(42)
“Healthy”	163	(33)	341	(70)	504	(51)
Unknown	29	(6)	38	(8)	67	(7)

a N/A, not available; PPU, pig production unit.

### 3.2 Virus detection and spatial analysis

Influenza A virus in pigs was detected in all departments in both years, with the exception of two departments each year (Tables S3 and S4). At the animal level, the percentage of positive pigs was 15.7% (CI_95%_: 12.4‐18.8, n=78/498) in 2010 and 11.7% (CI_95%_: 8.8‐14.6, n=57/487) in 2011. In 2010, detection of IAV by RT‐PCR was found to be associated with animals reported without apparent clinical signs at the time of sampling—herein referred to as “healthy” animals [OR_95%_=2.3 (1.4, 3.7), *P*=.0012]. Although the same association was not significant in 2011, when the information for health status was combined for both years, detection of IAV by RT‐PCR was significantly associated with sampling of “healthy” animals [OR_95%_=2.4 (1.4, 4.1), *P*=.001]. No association was found between IAV detection and type of PPU in either 2010 or 2011. Information for age and sex was only available for 2011 in which a borderline association was found between IAV detection and juvenile animals [OR_95%_=1.5 (0.7, 3.4)]. No association between IAV detection and sex was found. In summary, the risk factors at the animal level identified to be potentially associated with IAV detection in Guatemala were “healthy” and juvenile pigs (Table 2).

**Table 2:** Prevalence risk ratios of herd and animal risk factors associated with IAV detection by rRT‐PCR in sampled pigs in Guatemala, 2010‐2011

Variable	IAV positive (%)	IAV negative (%)	OR (95% CI)	*P*‐value
Herd level				
Type of PPU				
2010				
Backyard	22 (38.6)	79 (61.2)	Referent	
Farm	35 (61.4)	50 (38.8)	2.5 (1.3, 4.8)	.0047
2011				
Backyard	30 (36.2)	111 (75.5)	Referent	
Farm	17 (63.8)	36 (24.5)	1.7 (0.9, 3.5)	.1202
Total				
Backyard	52 (50.0)	190 (68.8)	Referent	
Farm	52 (50.0)	86 (31.2)	2.1 (1.3, 3.4)	.0016
Animal level				
Health status				
2010				
Sick	29 (39.7)	277 (70.0)	Referent	
“Healthy”	44 (60.3)	119 (30.0)	3.2 (1.7, 5.8)	.0001
2011				
Sick	11 (22.0)	97 (24.3)	Referent	
“Healthy”	39 (78.0)	302 (75.7)	1.1 (0.6, 2.3)	.7211
Total				
Sick	40 (32.5)	374 (47.1)		
“Healthy”	83 (67.5)	420 (52.9)	2.4 (1.4, 4.1)	.001
Sex^a^				
Female	23 (44.2)	165 (42.3)	Referent	
Male	29 (55.8)	225 (57.7)	0.9 (0.5, 1.7)	.8043
Age^a^				
Weanling (4‐10 wk)	8 (15.7)	80 (19.8)	Referent	
Juvenile (11‐17 wk)	20 (39.2)	132 (32.6)	1.6 (0.6, 4)	.3190
Semiadult (18‐20 wk)	5 (9.8)	40 (9.9)	1.2 (0.3, 4.8)	.7888
Adult (>5 wk)	18 (35.3)	153 (37.8)	1.1 (0.5, 2.9)	.7711

a Data available only for 2011. PPU, pig production unit.

At the herd level, the percentage of positive herds was 30.6% (CI_95%_: 24.0‐37.3, n=57/186) in 2010 and 24.2% (CI_95%_: 18.2‐30.3, n=47/194) in 2011, with no significant difference between the 2 years (Table S4). In 2010, the percentage of positive herds was higher for commercial farms [41.7%(CI_95%_: 30.7‐51.6)] in comparison with backyard units [21.8% (CI_95%_: 13.7‐29.8)] with a significant association between commercial farms and IAV detection [OR_95%_=2.5 (1.3, 4.8), *P*=.0048]. In 2011, the percentage of positive herds was also higher (but not significant) in commercial farms 32.1% (CI_95%_: 19.5‐44.6) when compared to backyard units 21.3% (CI_95%_: 14.5‐28.0). A borderline association between IAV detection and commercial farms [OR_95%_=1.7 (0.9, 3.5)] was observed. When data for type of PPU were combined for both years, commercial farms were more likely to be IAV positive than backyard units [OR_95%_=2.1 (1.3, 3.4), *P*=.002].

**Figure 1:**
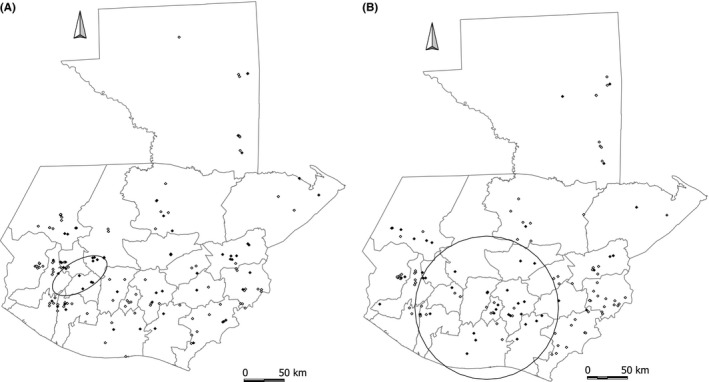
Spatial clusters of IAV‐positive farms obtained for (A) 2010 and (B) 2011 using the spatial scan statistic using an elliptical window, a maximum scanning window size of 50% of the population at risk, and 999 Monte Carlo randomizations. The ellipses represent the most likely clusters. Positive herds for IAV (rRT‐PCR) are shown in black squares and negative herds are shown in white. Pig densities per state are shown in Figure S2 as a reference

Herd spatial clusters were located and tested by scan statistics. No space–time clusters were observed; therefore, purely spatial analysis was performed for each year. In 2010, one cluster (*P*=.057) was observed located in the western part of the country (Table 3 and Figure 1, panel A). The length of the longest axis of the ellipse is 37.9 km and it comprises 12 herds, from which 11 were positive for IAV. In 2011, a major cluster was found (*P*=.0075), located in an area that included at least 13 departments. This cluster had a circular shape with a diameter of 89.98 km and comprised 43 herds, from which 27 were positive for IAV (Table 3 and Figure 1, panel B).

**Table 3:** Properties of spatial IAV clusters found using the scan statistic

Cluster	sma^a^ length (km)	smi^b^ length (km)	Population	Cases	*P*‐value	Relative risk
2010						
Most likely cluster	37.87	18.93	12	11	.057	2.95
2011						
Most likely cluster	89.98	89.98	43	27	.0075	3.00

a Semimajor axis.

b Semiminor axis.

### 3.3 Serologic detection and HI assay

With respect to IAV seroprevalence, 10.6% (CI_95%_: 7.9‐13.3) pigs were seropositive in 2010 and 1.4% (CI_95%_: 0.4‐2.4) in 2011. The association between pigs from commercial farms and antibody detection was also tested at the animal level. This association was significant in 2010 [OR_95%_=2.7 (1.2, 6.1)]. In 2011, the number of seropositive pigs was too low to compute associations with any of the other risk factors (type of PPU, age or sex). The percentage of seropositive herds was 16.1% (CI_95%_: 10.8‐21.4) and 3.1% (CI_95%_: 0.7‐5.5) for 2010 and 2011, respectively. In 2010, a higher number of seropositive herds were commercial farms [25.9% (CI_95%_: 16.6‐35.2)] in comparison with backyard units [7.9% (CI_95%_: 2.6‐13.2)]; and a significant association between commercial farms and detection of antibodies against IAV by ELISA was found [OR_95%_=4.1 (1.7, 9.7), *P*=.0016]. In 2011, the number of seropositive herds was too low to allow statistical comparisons at the herd level. In summary, commercial farms were associated with higher detection of antibodies against IAV.

Serological exposure to different subtypes varied between types of PPU: Exposure to H1 viruses of swine origin (including the α and ɣ clusters) was detected in commercial farms, whereas in backyards only exposure to pandemic H1 was found. Exposure to H3 clusters III and IV was detected in commercial farms in 2010 as single or multiple exposures. In backyards, exposure to H3 viruses was only found in samples with co‐exposure to pandemic H1 (Table 4).

**Table 4:** Antigenic responses detected by hemagglutination inhibition assay detected in pig sera, Guatemala, 2010‐2011

Virus (strain)	2010			2011		
	Farm (%)	Backyard (%)	Total (%)	Farm (%)	Backyard (%)	Total (%)
H1 pandemic (A/Mexico/4108/2009)	9 (20.5)	1 (11.1)	10 (18.9)	2 (66.7)	1 (25)	3 (42.9)
H1N1 α only (A/swine/MN/02053/2008)	5 (11.4)		5 (9.4)			
H1N1 β only (A/swine/NE/02013/2008)						
H1N1 γ only (A/swine/MO/02060/2008)	2 (4.5)		2 (3.8)			
H1N2 δ only (A/swine/IA/02039/2008)						
swH3N2 (III) only (A/swine/WI/14094/99)	9 (20.5)		9 (17)			
swH3 (IV) (A/swine/IL/A01201606/2011)	6 (13.6)		6 (11.3)			
huH3 only (A/swine/Guatemala/IP‐04‐0078/2008)						
pH1, H1 α		1 (11.1)	1 (1.9)			
pH1, H1 β	1 (2.3)		1 (1.9)			
pH1, H1 γ	1 (2.3)	2 (22.2)	3 (5.7)			
pH1, swH3 (III)		1 (11.1)	1 (1.9)			
pH1, swH3 (IV)	2 (4.5)		2 (3.8)		2 (50)	2 (28.6)
H1 γ, H3 (III)	1 (2.3)		1 (1.9)			
swH1	2 (4.5)	1 (11.1)	3 (5.7)			
swH1, H3 (III)	1 (2.3)		1 (1.9)			
U	5 (11.4)	3 (33.3)	8 (15.1)	1 (33.3)	1 (25)	2 (28.6)
Total^a^	44	9	53	3	4	7

a Number of HI positives from ELISA‐positive samples. Hu, human; sw, swine; p: 2009 pandemic strain.

### 3.4 Virus isolation, sequence characterization, and phylogenetic analysis

Four viruses were isolated from rRT‐PCR‐positive samples collected in 2010 (GenBank accessions KJ175112 to KJ175143). The viruses were identified as pandemic H1N1 (pH1N1, three isolates) and H3N2 (one isolate) subtypes based on >95% nucleotide sequence homology in BLAST searches. All gene segments of the three H1N1 viruses shared >98% sequence identity with each other and with the pandemic lineage. The remaining isolate, A/swine/Guatemala/CIP049‐IP040078/2010 (H3N2) (hereafter 040078‐H3N2), was identified as a fully human‐like strain. No reassortants were detected.

The pH1N1 isolates contained the prototypic motifs described for pandemic pH1N1 strains.^24^ These viruses were further analyzed for the presence of mutations in comparison with other viruses circulating in the region (Mexico, Central America, and the Caribbean) between 2009 and 2011. A number of mutations were identified in several gene segments (Table S5), including two non‐synonymous mutations in the HA (V251L and R222K), one in the PA (E688G), and two in the NA (G298A and a mixed base E462D). All of these mutations correspond to residues that are more frequently found in H1 viruses of swine origin. A specific role for these residues has not been described; the H1 HA R222K mutation lies within the antigenic site Ca1, while the others are mapped to experimentally determined epitopes reported in the IRD. The H1 HA protein contains five predicted glycosylation sites at amino acid positions 28, 40, 104, 294, and 304.

**Figure 2:**
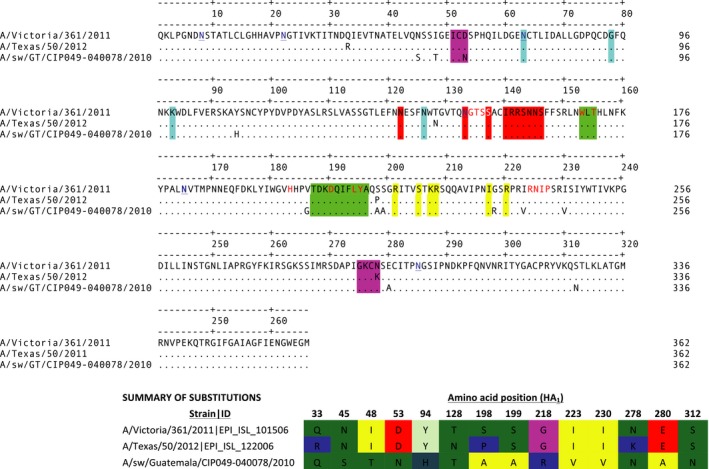
Alignment of the HA1 region of 040078‐H3N2 and current vaccine strains. Antigenic sites are shown in colored boxes: A (red), B (green), C (purple), D (yellow), E (blue). The N residues of predicted glycosylation sites for A/swine/Guatemala/CIP049‐040078/2010 are marked in blue and the receptor binding sites are marked in red in the reference sequence. Summary of the 14 amino acid substitutions is shown in the bottom left [Colour figure can be viewed at wileyonlinelibrary.com]

Analysis of phenotypic markers showed that 040078‐H3N2 is a well‐adapted human virus containing several markers associated with high transmissibility in humans.^25^ Seven glycosylation sites were predicted for the H3 HA protein at positions 24, 38, 79, 149, 181, 301, and 499. Protein alignments of the HA1 (H3) showed two mutations in the antigenic site C of the 040078‐H3N2 virus and at least 10 additional mutations when compared to the seasonal vaccine strains (Table S6, Figure 2).

**Figure 3:**
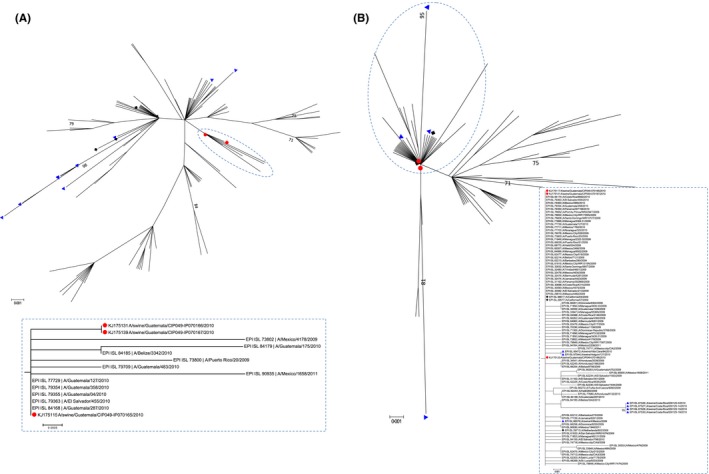
Phylogenetic inference for pH1N1 from Central America and the Caribbean (2009‐2013). (A) H1 hemagglutinin and (B) N1 neuraminidase. Maximum‐likelihood phylogenetic inference using the best‐fit model Hasegawa‐Kishino‐Yano (HKY) + Γ model of nucleotide substitution. Neighbor‐joining bootstrap support values ≥70% are shown. Reference strains, A/California/04/2009 (CA/04) and A/Netherlands/602/2009 (Nd/602), were included and are marked with black squares. The swine isolate from Guatemala is marked with a red circle. Swine isolates from other countries (Cuba, Mexico, and Costa Rica) are marked with blue triangles. The clusters delineated by a circle are shown at the bottom [Colour figure can be viewed at wileyonlinelibrary.com]

**Figure 4:**
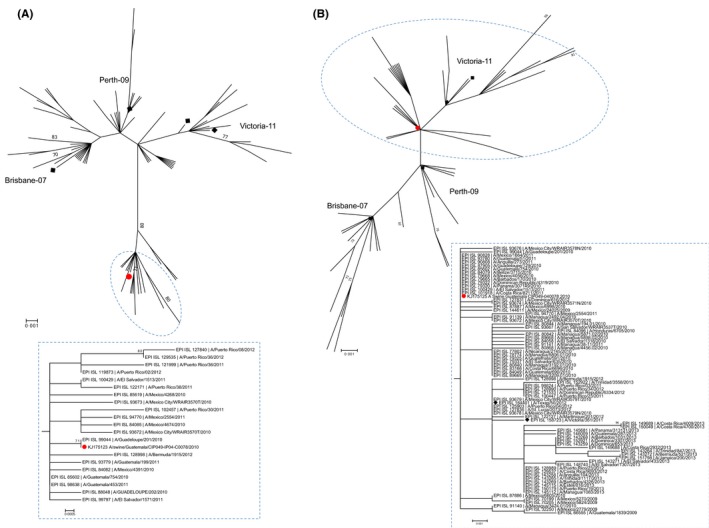
Maximum‐likelihood phylogenetic inference of the (A) hemagglutinin gene and (B) the neuraminidase gene of H3N2 human viruses from Central America and the Caribbean (2007‐2013), using the best‐fit model of nucleotide substitution, Hasegawa‐Kishino‐Yano (HKY) + GAMMA. Neighbor‐joining bootstrap support ≥70% values are shown. Reference vaccine strains were included to define lineages and are marked with black squares. The swine isolate from Guatemala is marked with a red circle. The clusters delineated by the circle are shown at the bottom. Scale bar depicts number of substitutions per site [Colour figure can be viewed at wileyonlinelibrary.com]

In the phylogenetic analysis, the Guatemalan pH1N1 swine isolates clustered with other contemporary human viruses from Guatemala, although in a separate cluster from that of the early human pH1N1 isolates and of other swine pH1N1 viruses from other neighboring countries, indicating independent introduction from humans (Figure 3, panels A and B). Short branch lengths were observed for the HA and the NA segments, suggesting that this was a recent introduction to pigs. For 040078‐H3N2 isolate, the HA and NA clustered with contemporary viruses that circulated in humans in Guatemala between 2010 and 2012 (Figure 3, panels A and B). However, for the HA, the virus clustered with other human viruses but in a separate clade from the most recent vaccine lineages (Figure 4, panel A). To analyze whether the circulation of viruses from this distinct clade was specific to Central America, background viruses from other geographic locations (North and South America, Africa, Asia, Europe, and Oceania) between 2007 and 2014 were included to construct a global phylogeny (Figure S1). In this tree, the swine isolate from Guatemala still clustered in a separate clade from the vaccine lineages but with viruses from other geographic regions, indicating that the circulation of viruses from this clade was not exclusive to Central America. Inclusion of HA sequences of recent human‐like swine isolates 26 revealed that viruses from the same clade as the 040078‐H3N2 have been recently introduced in pigs in the United States.

## 4 Discussion

The results of this study confirm the presence of IAV in the swine population in Guatemala between 2010 and 2011. An accurate estimation of IAV prevalence and seroprevalence from the data collected in this study is limited by the sampling approach. A more accurate estimation of prevalence would require sampling at the farm level to test for disease‐free status; here, samples were collected from individual pigs, distributed among the recruited PPUs regardless of herd size. Consequently, it is expected that the overall percentage of positive and seropositive herds found in this study underestimates IAV prevalence. Nevertheless, the detection rates fall within the ranges reported for other countries in the region.^27^, ^28^, ^29^ Detection by rRT‐PCR yielded 14% of positive samples over the 2‐year period, with more than 30% of positive herds each year. In contrast, relatively low seropositivity was found. The lower exposure observed in 2011 compared to 2010 may indicate variations in the levels of IAV infection in pigs throughout the year, as samples were taken during different months each year. Moreover, the potentially less‐than‐optimal capacity of the diagnostic test utilized to detect locally circulating strains could account for the low numbers of seropositive animals observed. Our results suggest that commercial farms are at higher risk of IAV exposure. Similar to other regions, in Guatemala, confinement of animals and increased population densities related to commercial farming practices seem to be important in the epidemiology of swine IAV. Other environmental factors that influence IAV transmission in tropical countries like Guatemala are not well defined; in humans the proportion of IAV cases usually reaches a peak during the rainy season (from May to August) and IAV transmission seem to be associated with specific humidity.^30^, ^31^, ^32^ Similar to humans, seasonal infections of IAV in backyard pigs are plausible, whereas in commercial farms, IAV infection may be directly linked to nature of the pig production cycle. Longitudinal studies are necessary to further address these questions.

In 2010, the majority of samples were collected from pigs reported with signs of respiratory disease (regardless of type of PPU); remarkably, most of the positive samples were from pigs reported as “healthy” at the time of sampling. In 2011, the majority of sampled pigs were reported as “healthy” and similar levels of detection were observed in “healthy” and sick animals. The relatively high number of positive “healthy” animals observed may be partly explained by the occurrence of subclinical infections.^33^ However, the information collected on health status may be biased by the ability of the collector to observe respiratory signs at the time of sampling. In terms of IAV detection by age, higher risk of IAV infection observed in juvenile pigs is in agreement with other studies.^34^, ^35^ As for virus exposure, only a number of seropositive animals were detected in 2011, and expectedly, most of the samples were from sows. The HI results indicate that backyard pigs may be at higher risk of exposure to viruses of human origin, whereas pigs in commercial farms may be at higher risk of exposure to swine‐derived strains associated with animal movement. Serological exposure to viruses of different origin reflects differences in the epidemiology of IAV between these two populations. Interestingly, exposure to the pH1N1 virus was found in the majority of positive animals, regardless of the type of PPU. Introduction of pandemic viruses into pigs has been documented in other countries in Central America and the Caribbean.^27^, ^28^, ^36^ In Guatemala, reverse zoonotic transmission from humans is the most likely explanation to the origin of the pH1N1 viruses circulating in pigs. However, in the absence of information about the viruses that circulated in pigs prior to the emergence of the pandemic, circulation of other H1 viruses with similar antigenic properties to the pH1N1 cannot be ruled out. Multiple exposures (i.e, HI reactivity to more than one virus) were detected in a number of samples, suggesting circulation of different viruses in positive PPUs. In general, the HI titers against the tested antigens were relatively low (geometric mean titers, GMT, ranged between 80 and 269, data not shown), which may also indicate cross‐reactive rather than strain‐specific responses. Failure to identify the specific response of 10 of the ELISA‐positive samples could be a sign that other viruses with different antigenicity circulate in pigs in Guatemala. Inclusion of more recent strains in the diagnostics panel as well as continued characterization of local strains is crucial to improve current diagnostics. In conjunction with the HI results, the isolation of only strains of human origin suggests an important role of humans in the epidemiology of swine IAV in Guatemala. The human‐like H3N2 isolate was obtained from the department “El Progreso” where, according to data from the ministry of health, the reported incidence of acute respiratory infection in humans was higher than the national average in 2010.^37^ With the current amount of IAV isolates and epidemiologic information available from pigs and humans, it is hard to analyze the extent of cross‐species transmission between these hosts. Future studies should focus at the swine–human interface to address these questions.

For the spatial analysis, aggregation of positive herds detected by scan statistics could be explained by persistence of the virus over time by yet unknown mechanisms and spread to neighboring herds through animal movement or contaminated materials. In the case of backyard populations, we hypothesize that trade of animals among neighboring villages could serve as carriers for localized virus spread, whereas animal trade among commercial farms could serve as vehicles for virus spread at larger distances and to different departments. No space–time cluster was observed by spatial scan statistics. However, detection of space–time clustering is more likely when the information is collected over a longer time period, or with more frequency.^38^ With respect to the location of the clusters, the cluster observed in 2010 is located in the highlands (1000 to 3000 MASL) where annual average temperatures range between 7 and 22°C.^39^ The lower temperatures and relative humidity in this region, compared to other parts of the country, may facilitate transmission of IAVs in pigs. The cluster from 2011 includes the capital, Guatemala City, which, with around 5 000 000 inhabitants, is the most populated in the country. This same cluster also covered the departments of Santa Rosa, Jutiapa, and Escuintla, where low pathogenic avian influenza viruses (LPAIV) have been detected in migratory waterfowl and where a new lineage of IAV from bats was reported in 2010.^15^, ^40^ In Guatemala, the majority of herds are located near rural communities that rely on small‐scale farming as a source of income. Further studies at those locations could help to establish the levels of exposure of pigs to other IAV subtypes. Given the diversity of IAVs that has been detected in animal reservoirs in Guatemala, we recommend investigating outbreaks of IAV in swine and other domestic animals, as well as implementing educational programs in local communities to increase biosecurity in backyard units.

For the virus isolates, the presence of amino acid residues that are prevalent in other viruses of swine origin suggests undergoing adaptation of these viruses in the swine host. Although no reassortant viruses were found, introduction of the pH1N1 to pigs in Guatemala may represent the establishment of a novel genetic lineage with the potential to reassort with cocirculating viruses. Circulation of human H3N2 viruses from a distinct clade (defined as clade 6 by the WHO) has not been extensively documented.^41^, ^42^ In addition to the swine isolate, we found that the majority of H3 hemagglutinin sequences from Central America reported between 2010 and 2011, and some from 2012, were contained within this clade. The amount of data available for human influenza for Central America is extremely limited, and Central America is not considered a major contributor to the epidemiology of human influenza in a continental scale.^43^ A low connection of Central American countries by air travel 44 summed to differences in influenza seasonality in the Neotropics 31 may facilitate the circulation of virus populations genetically different in this region. The mutations in the HA1 protein of 040078‐H3N2 in comparison with the vaccine strains suggest differences in antigenicity with other viruses of human origin. Significant antigenic distance was shown for recent swine isolates from the same genetic cluster,^26^ meaning that even when other H3 viruses already circulate in pigs in Guatemala, there could be little cross‐protection to a virus like the 040078‐H3N2. Experimental evaluation of the transmissibility of this virus in pigs could help assess the risk of onward transmission in this host.

In summary, our results evidence that different IAVs circulate in the Guatemalan swine population in two production systems. Differences in virus detection, levels of exposure, and distribution of positive herds between years suggest temporal variation in the circulation of influenza that warrants further investigation with longitudinal studies. Although in commercial farms, “healthy” and juvenile pigs were factors found to be associated with IAV infection, these findings should be interpreted with caution and confirmed with appropriate studies. Lastly, our study provides evidence of the contribution of humans to the molecular epidemiology of IAV in swine in Guatemala and evidences gaps in current local animal and human surveillance. Our findings underscore the importance of continuing sampling to increase the number of virus isolates from the different host of IAV. This information is crucial to improve our understanding of the evolution and epidemiology of IAV in Guatemala and the Neotropics.
