# Supplementary material for: Origin, distribution, and potential risk factors associated with influenza A virus in swine in two production systems in Guatemala
Source: Influenza Other Respir Viruses. 2017 Jan 30;11(2):182–92. doi: 10.1111/irv.12437 (PMC5304577; doi:10.1111/irv.12437)
Supplement: Supplementary file 3 [file IRV-11-182-s003.docx]

**Table S1**. Sequences used for phylogenetic analysis of the swine influenza A viruses from Guatemala and retrieved from GISAID. We acknowledge the authors, originating and submitting laboratories of the sequences from GISAID’s EpiFlu™ Database that were used as background data for generating the phylogenetic trees of the virus isolated from Guatemala. The list is detailed below.

| Segment ID | Segment | Country | Collection date | Isolate name | Originating Lab | Submitting Lab |
| --- | --- | --- | --- | --- | --- | --- |
| EPI216724 | HA (H1) | Barbados | 2009-Sep-04 | A/Barbados/275/2009 | Public Health Laboratory | Centers for Disease Control and Prevention |
| EPI211402 | HA (H1) | Barbados | 2009-Sep-07 | A/Barbados/7992/2009 | Caribbean Epidemiology Center | Centers for Disease Control and Prevention |
| EPI216727 | HA (H1) | Barbados | 2009-Sep-10 | A/Barbados/280/2009 | Public Health Laboratory | Centers for Disease Control and Prevention |
| EPI319999 | HA (H1) | Barbados | 2011-Jan-12 | A/Barbados/104/2011 | Caribbean Epidemiology Center | Centers for Disease Control and Prevention |
| EPI497720 | HA (H1) | Barbados | 2013-Sep-19 | A/Barbados/3024/2013 | Caribbean Epidemiology Center | Centers for Disease Control and Prevention |
| EPI216730 | HA (H1) | Belize | 2009-Jul-28 | A/Belize/7121/2009 | Caribbean Epidemiology Center | Centers for Disease Control and Prevention |
| EPI231291 | HA (H1) | Belize | 2009-Oct-08 | A/Belize/8756/2009 | Caribbean Epidemiology Center | Centers for Disease Control and Prevention |
| EPI295492 | HA (H1) | Belize | 2010-Aug-11 | A/Belize/3342/2010 | Caribbean Epidemiology Center | Centers for Disease Control and Prevention |
| EPI394847 | HA (H1) | Belize | 2012-Jan-01 | A/Belize/334/2012 | Caribbean Epidemiology Center | Centers for Disease Control and Prevention |
| EPI497711 | HA (H1) | Belize | 2013-Sep-23 | A/Belize/3161/2013 | Caribbean Epidemiology Center | Centers for Disease Control and Prevention |
| EPI186269 | HA (H1) | Bermuda | 2009-Jun-03 | A/Bermuda/4287/2009 | Caribbean Epidemiology Center | Centers for Disease Control and Prevention |
| EPI220980 | HA (H1) | British Virgin Islands | 2009-Jun-15 | A/British Virgin Islands/5437/2009 | Caribbean Epidemiology Center | Centers for Disease Control and Prevention |
| EPI497726 | HA (H1) | British Virgin Islands | 2013-Oct-01 | A/British Virgin Islands/3138/2013 | Caribbean Epidemiology Center | Centers for Disease Control and Prevention |
| EPI320005 | HA (H1) | Cayman Islands | 2011-Jan-14 | A/Cayman Islands/284/2011 | Caribbean Epidemiology Center | Centers for Disease Control and Prevention |
| EPI181744 | HA (H1) | Costa Rica | 2009-Apr-28 | A/Costa Rica/4314/2009 | Laboratorio Nacional de Influenza | Centers for Disease Control and Prevention |
| EPI227650 | HA (H1) | Costa Rica | 2009-Jul-13 | A/Costa Rica/3149/2009 | Laboratorio Nacional de Influenza | Centers for Disease Control and Prevention |
| EPI216767 | HA (H1) | Costa Rica | 2009-Jul-17 | A/Costa Rica/3896/2009 | Laboratorio Nacional de Influenza | Centers for Disease Control and Prevention |
| EPI185355 | HA (H1) | Costa Rica | 2009-May-02 | A/Costa Rica/4857/2009 |  | Centers for Disease Control and Prevention |
| EPI216778 | HA (H1) | Costa Rica | 2009-May-16 | A/Costa Rica/6321/2009 | Laboratorio Nacional de Influenza | Centers for Disease Control and Prevention |
| EPI295456 | HA (H1) | Costa Rica | 2010-Jul-16 | A/Costa Rica/6692/2010 | Laboratorio Nacional de Influenza | Centers for Disease Control and Prevention |
| EPI295450 | HA (H1) | Costa Rica | 2010-May-06 | A/Costa Rica/5458/2010 | Laboratorio Nacional de Influenza | Centers for Disease Control and Prevention |
| EPI295453 | HA (H1) | Costa Rica | 2010-May-07 | A/Costa Rica/5529/2010 | Laboratorio Nacional de Influenza | Centers for Disease Control and Prevention |
| EPI307811 | HA (H1) | Costa Rica | 2010-Nov-01 | A/swine/Costa Rica/000125-3/2010 |  | Other Database Import |
| EPI307814 | HA (H1) | Costa Rica | 2010-Nov-01 | A/swine/Costa Rica/000125-14/2010 |  | Other Database Import |
| EPI307817 | HA (H1) | Costa Rica | 2010-Nov-01 | A/swine/Costa Rica/000125-15/2010 |  | Other Database Import |
| EPI307820 | HA (H1) | Costa Rica | 2010-Nov-01 | A/swine/Costa Rica/000125-16/2010 |  | Other Database Import |
| EPI307823 | HA (H1) | Costa Rica | 2010-Nov-01 | A/swine/Costa Rica/000125-19/2010 |  | Other Database Import |
| EPI307826 | HA (H1) | Costa Rica | 2010-Nov-01 | A/swine/Costa Rica/000125-20/2010 |  | Other Database Import |
| EPI350003 | HA (H1) | Costa Rica | 2011-Oct-11 | A/Costa Rica/8107/2011 | Laboratorio Nacional de Influenza | Centers for Disease Control and Prevention |
| EPI348171 | HA (H1) | Costa Rica | 2011-Oct-30 | A/Costa Rica/6796/2011 | Laboratorio Nacional de Influenza | Centers for Disease Control and Prevention |
| EPI376365 | HA (H1) | Costa Rica | 2012-Jan-17 | A/Costa Rica/4023/2012 | Laboratorio Nacional de Influenza | Centers for Disease Control and Prevention |
| EPI397472 | HA (H1) | Costa Rica | 2012-Jun-24 | A/Costa Rica/6288/2012 | Laboratorio Nacional de Influenza | Centers for Disease Control and Prevention |
| EPI482164 | HA (H1) | Costa Rica | 2013-Jun-18 | A/Costa Rica/8288/2013 | Laboratorio Nacional de Influenza | Centers for Disease Control and Prevention |
| EPI482166 | HA (H1) | Costa Rica | 2013-May-30 | A/Costa Rica/5790/2013 | Laboratorio Nacional de Influenza | Centers for Disease Control and Prevention |
| EPI335977 | HA (H1) | Cuba | 2010-Nov-01 | A/swine/Villa Clara/84/2010 |  | Other Database Import |
| EPI338288 | HA (H1) | Cuba | 2010-Nov-01 | A/swine/Pinar del Rio/3/2010 |  | Other Database Import |
| EPI338314 | HA (H1) | Cuba | 2010-Nov-01 | A/swine/La Habana/130/2010 |  | Other Database Import |
| EPI247263 | HA (H1) | Dominican Republic | 2009-Dec-15 | A/Dominican Republic/3768/2009 | Laboratorio de Investigacion / Centro de Educacion Medica y Amistad Dominico Japones (CEMADOJA) | Centers for Disease Control and Prevention |
| EPI215438 | HA (H1) | Dominican Republic | 2009-Jul-02 | A/Santo Domingo/WR1057N/2009 |  | Other Database Import |
| EPI215446 | HA (H1) | Dominican Republic | 2009-Jul-02 | A/Santo Domingo/WR1058N/2009 |  | Other Database Import |
| EPI277657 | HA (H1) | Dominican Republic | 2009-Jun-10 | A/Santo Domingo/WR1072T/2009 |  | Other Database Import |
| EPI215462 | HA (H1) | Dominican Republic | 2009-Jun-25 | A/Santo Domingo/WR1068N/2009 |  | Other Database Import |
| EPI215454 | HA (H1) | Dominican Republic | 2009-Jun-30 | A/Santo Domingo/WR1059N/2009 |  | Other Database Import |
| EPI186244 | HA (H1) | Dominican Republic | 2009-May-24 | A/Santo Domingo/572N/2009 |  | Other Database Import |
| EPI186657 | HA (H1) | Dominican Republic | 2009-May-26 | A/Santo Domingo/565T/2009 |  | Other Database Import |
| EPI231305 | HA (H1) | Dominican Republic | 2009-Oct-23 | A/Dominica/9250/2009 | Caribbean Epidemiology Center | Centers for Disease Control and Prevention |
| EPI320014 | HA (H1) | Dominican Republic | 2011-Apr-11 | A/Dominican Republic/5145/2011 | Laboratorio de Investigacion / Centro de Educacion Medica y Amistad Dominico Japones (CEMADOJA) | Centers for Disease Control and Prevention |
| EPI335804 | HA (H1) | Dominican Republic | 2011-Jul-18 | A/Dominican Republic/5542/2011 | Laboratorio de Investigacion / Centro de Educacion Medica y Amistad Dominico Japones (CEMADOJA) | Centers for Disease Control and Prevention |
| EPI320011 | HA (H1) | Dominican Republic | 2011-Mar-28 | A/Dominican Republic/5059/2011 | Laboratorio de Investigacion / Centro de Educacion Medica y Amistad Dominico Japones (CEMADOJA) | Centers for Disease Control and Prevention |
| EPI376381 | HA (H1) | Dominican Republic | 2012-Feb-21 | A/Dominican Republic/6188/2012 | Laboratorio de Investigacion / Centro de Educacion Medica y Amistad Dominico Japones (CEMADOJA) | Centers for Disease Control and Prevention |
| EPI395238 | HA (H1) | Dominican Republic | 2012-Jun-08 | A/Dominican Republic/6551/2012 | Laboratorio de Investigacion / Centro de Educacion Medica y Amistad Dominico Japones (CEMADOJA) | Centers for Disease Control and Prevention |
| EPI396139 | HA (H1) | Dominican Republic | 2012-May-28 | A/Dominican Republic/6493/2012 | Laboratorio de Investigacion / Centro de Educacion Medica y Amistad Dominico Japones (CEMADOJA) | Centers for Disease Control and Prevention |
| EPI459759 | HA (H1) | Dominican Republic | 2013-Apr-01 | A/Dominican Republic/7238/2013 | Laboratorio de Investigacion / Centro de Educacion Medica y Amistad Dominico Japones (CEMADOJA) | Centers for Disease Control and Prevention |
| EPI484726 | HA (H1) | Dominican Republic | 2013-Jun-05 | A/Dominican Republic/7548/2013 | Laboratorio de Investigacion / Centro de Educacion Medica y Amistad Dominico Japones (CEMADOJA) | Centers for Disease Control and Prevention |
| EPI467200 | HA (H1) | Dominican Republic | 2013-Jun-10 | A/Dominican Republic/7703/2013 | Laboratorio de Investigacion / Centro de Educacion Medica y Amistad Dominico Japones (CEMADOJA) | Centers for Disease Control and Prevention |
| EPI467203 | HA (H1) | Dominican Republic | 2013-Jun-12 | A/Dominican Republic/7603/2013 | Laboratorio de Investigacion / Centro de Educacion Medica y Amistad Dominico Japones (CEMADOJA) | Centers for Disease Control and Prevention |
| EPI477445 | HA (H1) | Dominican Republic | 2013-Jun-15 | A/Dominican Republic/7626/2013 | Laboratorio de Investigacion / Centro de Educacion Medica y Amistad Dominico Japones (CEMADOJA) | Centers for Disease Control and Prevention |
| EPI457456 | HA (H1) | Dominican Republic | 2013-May-01 | A/Dominican Republic/7291/2013 | Laboratorio de Investigacion / Centro de Educacion Medica y Amistad Dominico Japones (CEMADOJA) | Centers for Disease Control and Prevention |
| EPI457503 | HA (H1) | Dominican Republic | 2013-May-01 | A/Dominican Republic/7293/2013 | Laboratorio de Investigacion / Centro de Educacion Medica y Amistad Dominico Japones (CEMADOJA) | Centers for Disease Control and Prevention |
| EPI179074 | HA (H1) | El Salvador | 2009-Apr-26 | A/El Salvador/213/2009 | Contiguo a Hospital Rosales | Centers for Disease Control and Prevention |
| EPI215398 | HA (H1) | El Salvador | 2009-Jun-09 | A/San Salvador/WR0167N/2009 |  | Other Database Import |
| EPI215390 | HA (H1) | El Salvador | 2009-Jun-12 | A/San Salvador/0169T/2009 |  | Other Database Import |
| EPI183131 | HA (H1) | El Salvador | 2009-May-13 | A/El Salvador/351/2009 | Contiguo a Hospital Rosales | Centers for Disease Control and Prevention |
| EPI295399 | HA (H1) | El Salvador | 2010-Jul-13 | A/El Salvador/798/2010 | Contiguo a Hospital Rosales | Centers for Disease Control and Prevention |
| EPI278888 | HA (H1) | El Salvador | 2010-May-26 | A/El Salvador/455/2010 | Contiguo a Hospital Rosales | Centers for Disease Control and Prevention |
| EPI380014 | HA (H1) | El Salvador | 2012-Apr-17 | A/El Salvador/530/2012 | Contiguo a Hospital Rosales | Centers for Disease Control and Prevention |
| EPI484974 | HA (H1) | El Salvador | 2013-Jul-05 | A/El Salvador/1396/2013 | Contiguo a Hospital Rosales | Centers for Disease Control and Prevention |
| EPI534020 | HA (H1) | El Salvador | 2013-Nov-04 | A/El Salvador/2260/2013 | Contiguo a Hospital Rosales | Centers for Disease Control and Prevention |
| EPI508446 | HA (H1) | El Salvador | 2013-Nov-22 | A/El Salvador/2360/2013 | Contiguo a Hospital Rosales | Centers for Disease Control and Prevention |
| EPI231312 | HA (H1) | Grenada | 2009-Oct-23 | A/Grenada/9302/2009 | Caribbean Epidemiology Center | Centers for Disease Control and Prevention |
| EPI216851 | HA (H1) | Guatemala | 2009-Aug-03 | A/Guatemala/3798/2009 | Laboratorio Nacional De Salud Guatemala | Centers for Disease Control and Prevention |
| EPI216848 | HA (H1) | Guatemala | 2009-Jul-14 | A/Guatemala/3185/2009 | Laboratorio Nacional De Salud Guatemala | Centers for Disease Control and Prevention |
| EPI194155 | HA (H1) | Guatemala | 2009-Jun-02 | A/Guatemala/1039/2009 | Laboratorio Nacional De Salud Guatemala | Centers for Disease Control and Prevention |
| EPI231317 | HA (H1) | Guatemala | 2009-Oct-26 | A/Guatemala/4752/2009 | Laboratorio Nacional De Salud Guatemala | Centers for Disease Control and Prevention |
| EPI231315 | HA (H1) | Guatemala | 2009-Sep-15 | A/Guatemala/4360/2009 | Laboratorio Nacional De Salud Guatemala | Centers for Disease Control and Prevention |
| EPI278863 | HA (H1) | Guatemala | 2010-Apr-28 | A/Guatemala/358/2010 | Laboratorio Nacional De Salud Guatemala | Centers for Disease Control and Prevention |
| EPI295438 | HA (H1) | Guatemala | 2010-Aug-27 | A/Guatemala/287/2010 | Laboratorio Nacional De Salud Guatemala | Centers for Disease Control and Prevention |
| EPI273937 | HA (H1) | Guatemala | 2010-Jan-01 | A/Guatemala/127/2010 |  | Centers for Disease Control and Prevention |
| EPI278869 | HA (H1) | Guatemala | 2010-Jul-19 | A/Guatemala/598/2010 | Laboratorio Nacional De Salud Guatemala | Centers for Disease Control and Prevention |
| EPI280305 | HA (H1) | Guatemala | 2010-Jun-03 | A/Guatemala/483/2010 | Laboratorio Nacional De Salud Guatemala | Centers for Disease Control and Prevention |
| EPI278866 | HA (H1) | Guatemala | 2010-Jun-12 | A/Guatemala/04/2010 | Laboratorio Nacional De Salud Guatemala | Centers for Disease Control and Prevention |
| EPI295471 | HA (H1) | Guatemala | 2010-Sep-17 | A/Guatemala/175/2010 | Laboratorio Nacional De Salud Guatemala | Centers for Disease Control and Prevention |
| EPI362991 | HA (H1) | Guatemala | 2012-Feb-12 | A/Guatemala/18/2012 | Laboratorio Nacional De Salud Guatemala | Centers for Disease Control and Prevention |
| EPI216857 | HA (H1) | Haiti | 2009-Aug-12 | A/Haiti/265/2009 | Laboratoire National de Sante Publique | Centers for Disease Control and Prevention |
| EPI278009 | HA (H1) | Haiti | 2009-Nov-13 | A/Port Au Prince/WR2294T/2009 |  | Other Database Import |
| EPI239674 | HA (H1) | Haiti | 2009-Oct-19 | A/Haiti/534/2009 | Laboratoire National de Sante Publique | Centers for Disease Control and Prevention |
| EPI465088 | HA (H1) | Haiti | 2013-Jun-02 | A/Haiti/1790/2013 | Caribbean Epidemiology Center | Centers for Disease Control and Prevention |
| EPI465708 | HA (H1) | Haiti | 2013-Jun-02 | A/Haiti/1792/2013 | Caribbean Epidemiology Center | Centers for Disease Control and Prevention |
| EPI465403 | HA (H1) | Haiti | 2013-Jun-18 | A/Haiti/2030/2013 | Caribbean Epidemiology Center | Centers for Disease Control and Prevention |
| EPI465472 | HA (H1) | Haiti | 2013-Jun-20 | A/Haiti/2036/2013 | Caribbean Epidemiology Center | Centers for Disease Control and Prevention |
| EPI191948 | HA (H1) | Honduras | 2009-Jun-17 | A/Honduras/2039/2009 | Laboratorio Nacional de Virologia | Centers for Disease Control and Prevention |
| EPI216863 | HA (H1) | Honduras | 2009-Jun-25 | A/Honduras/2188/2009 | Laboratorio Nacional de Virologia | Centers for Disease Control and Prevention |
| EPI273771 | HA (H1) | Honduras | 2010-May-19 | A/Honduras/6132/2010 | Laboratorio Nacional de Virologia | Centers for Disease Control and Prevention |
| EPI394737 | HA (H1) | Honduras | 2012-Jun-14 | A/Honduras/9720/2012 | Laboratorio Nacional de Virologia | Centers for Disease Control and Prevention |
| EPI394852 | HA (H1) | Honduras | 2012-Jun-26 | A/Honduras/9746/2012 | Laboratorio Nacional de Virologia | Centers for Disease Control and Prevention |
| EPI273920 | HA (H1) | Jamaica | 2009-Jul-07 | A/Jamaica/6261/2009 | University of the West Indies | Centers for Disease Control and Prevention |
| EPI186266 | HA (H1) | Jamaica | 2009-Jun-06 | A/Jamaica/4423/2009 | Caribbean Epidemiology Center | Centers for Disease Control and Prevention |
| EPI391296 | HA (H1) | Jamaica | 2012-Feb-13 | A/Jamaica/764/2012 | University of the West Indies | Centers for Disease Control and Prevention |
| EPI497702 | HA (H1) | Jamaica | 2013-Oct-30 | A/Jamaica/198/2013 | University of the West Indies | Centers for Disease Control and Prevention |
| EPI189129 | HA (H1) | Martinique | 2009-Jun-20 | A/Martinique/15/2009 | National Influenza Center French Guiana and French Indies | Centers for Disease Control and Prevention |
| EPI230497 | HA (H1) | Mexico | 2009-Apr-01 | A/swine/4/Mexico/2009 |  | Other Database Import |
| EPI177338 | HA (H1) | Mexico | 2009-Apr-03 | A/Mexico/4108/2009 | Laboratorio de Virus Respiratorio | Centers for Disease Control and Prevention |
| EPI179080 | HA (H1) | Mexico | 2009-Apr-03 | A/Mexico/3955/2009 | Laboratorio de Virus Respiratorio | Centers for Disease Control and Prevention |
| EPI176587 | HA (H1) | Mexico | 2009-Apr-14 | A/Mexico/4482/2009 | Laboratorio de Virus Respiratorio | Centers for Disease Control and Prevention |
| EPI179102 | HA (H1) | Mexico | 2009-Apr-20 | A/Mexico/4593/2009 | Laboratorio de Virus Respiratorio | Centers for Disease Control and Prevention |
| EPI180755 | HA (H1) | Mexico | 2009-Apr-25 | A/Mexico/47N/2009 |  | Other Database Import |
| EPI190863 | HA (H1) | Mexico | 2009-Apr-25 | A/Mexico/48N/2009 |  | Other Database Import |
| EPI266952 | HA (H1) | Mexico | 2009-Apr-30 | A/Mexico City/020/2009 |  | Other Database Import |
| EPI273882 | HA (H1) | Mexico | 2009-Dec-18 | A/Mexico/5569/2009 | Laboratorio de Virus Respiratorio | Centers for Disease Control and Prevention |
| EPI179001 | HA (H1) | Mexico | 2009-Jan-01 | A/Mexico/InDRE4114/2009 |  | Other Database Import |
| EPI231329 | HA (H1) | Mexico | 2009-Jul-10 | A/Mexico/2466/2009 | Laboratorio de Virus Respiratorio | Centers for Disease Control and Prevention |
| EPI215270 | HA (H1) | Mexico | 2009-Jun-19 | A/Mexico City/WR1087T/2009 |  | Other Database Import |
| EPI215278 | HA (H1) | Mexico | 2009-Jun-29 | A/Mexico City/WR1100N/2009 |  | Other Database Import |
| EPI231326 | HA (H1) | Mexico | 2009-Jun-30 | A/Mexico/2445/2009 | Laboratorio de Virus Respiratorio | Centers for Disease Control and Prevention |
| EPI232961 | HA (H1) | Mexico | 2009-Mar-12 | A/Mexico/2964/2009 | Laboratorio de Virus Respiratorio | Centers for Disease Control and Prevention |
| EPI266992 | HA (H1) | Mexico | 2009-May-01 | A/Mexico City/025/2009 |  | Other Database Import |
| EPI267000 | HA (H1) | Mexico | 2009-May-02 | A/Mexico City/026/2009 |  | Other Database Import |
| EPI217490 | HA (H1) | Mexico | 2009-May-07 | A/Mexico City/001/2009 |  | Other Database Import |
| EPI217570 | HA (H1) | Mexico | 2009-May-09 | A/Mexico City/011/2009 |  | Other Database Import |
| EPI217578 | HA (H1) | Mexico | 2009-May-09 | A/Mexico City/012/2009 |  | Other Database Import |
| EPI217594 | HA (H1) | Mexico | 2009-May-09 | A/Mexico City/015/2009 |  | Other Database Import |
| EPI217546 | HA (H1) | Mexico | 2009-May-10 | A/Mexico City/008/2009 |  | Other Database Import |
| EPI217610 | HA (H1) | Mexico | 2009-May-10 | A/Mexico City/018/2009 |  | Other Database Import |
| EPI256738 | HA (H1) | Mexico | 2009-May-16 | A/Mexico city/CIA9/2009 |  | Other Database Import |
| EPI277977 | HA (H1) | Mexico | 2009-Nov-09 | A/Mexico City/WR1747N/2009 |  | Other Database Import |
| EPI243905 | HA (H1) | Mexico | 2009-Nov-18 | A/Mexico/476/2009 | Laboratorio de Virus Respiratorio | Centers for Disease Control and Prevention |
| EPI277993 | HA (H1) | Mexico | 2009-Nov-19 | A/Mexico City/WR1765N/2009 |  | Other Database Import |
| EPI277921 | HA (H1) | Mexico | 2009-Oct-01 | A/Mexico City/WR1695N/2009 |  | Other Database Import |
| EPI277881 | HA (H1) | Mexico | 2009-Oct-05 | A/Mexico City/WR1673N/2009 |  | Other Database Import |
| EPI277969 | HA (H1) | Mexico | 2009-Oct-09 | A/Mexico City/WR1708T/2009 |  | Other Database Import |
| EPI273925 | HA (H1) | Mexico | 2009-Oct-18 | A/Mexico/4178/2009 | Laboratorio de Virus Respiratorio | Centers for Disease Control and Prevention |
| EPI277665 | HA (H1) | Mexico | 2009-Oct-22 | A/Mexico City/WR1090N/2009 |  | Other Database Import |
| EPI243933 | HA (H1) | Mexico | 2009-Oct-26 | A/Mexico/1138/2009 | Laboratorio de Virus Respiratorio | Centers for Disease Control and Prevention |
| EPI215302 | HA (H1) | Mexico | 2009-Sep-02 | A/Mexico City/WR1306N/2009 |  | Other Database Import |
| EPI215286 | HA (H1) | Mexico | 2009-Sep-03 | A/Mexico City/WR1297N/2009 |  | Other Database Import |
| EPI215326 | HA (H1) | Mexico | 2009-Sep-09 | A/Mexico City/WR1310N/2009 |  | Other Database Import |
| EPI215334 | HA (H1) | Mexico | 2009-Sep-10 | A/Mexico City/WR1311T/2009 |  | Other Database Import |
| EPI215294 | HA (H1) | Mexico | 2009-Sep-12 | A/Mexico City/WR1301N/2009 |  | Other Database Import |
| EPI215310 | HA (H1) | Mexico | 2009-Sep-14 | A/Mexico City/WR1307N/2009 |  | Other Database Import |
| EPI277937 | HA (H1) | Mexico | 2009-Sep-15 | A/Mexico City/WR1697T/2009 |  | Other Database Import |
| EPI277929 | HA (H1) | Mexico | 2009-Sep-17 | A/Mexico City/WR1696T/2009 |  | Other Database Import |
| EPI277961 | HA (H1) | Mexico | 2009-Sep-18 | A/Mexico City/WR1706T/2009 |  | Other Database Import |
| EPI277873 | HA (H1) | Mexico | 2009-Sep-23 | A/Mexico City/WR1668T/2009 |  | Other Database Import |
| EPI277889 | HA (H1) | Mexico | 2009-Sep-23 | A/Mexico City/WR1675T/2009 |  | Other Database Import |
| EPI277905 | HA (H1) | Mexico | 2009-Sep-23 | A/Mexico City/WR1687T/2009 |  | Other Database Import |
| EPI278878 | HA (H1) | Mexico | 2010-Apr-07 | A/Mexico/2880/2010 | Laboratorio de Virus Respiratorio | Centers for Disease Control and Prevention |
| EPI273879 | HA (H1) | Mexico | 2010-Mar-01 | A/Mexico/1762/2010 | Laboratorio de Virus Respiratorio | Centers for Disease Control and Prevention |
| EPI355516 | HA (H1) | Mexico | 2011-Dec-12 | A/Mexico/3752/2011 | Laboratorio de Virus Respiratorio | Centers for Disease Control and Prevention |
| EPI353405 | HA (H1) | Mexico | 2011-Dec-14 | A/Mexico/3720/2011 | Laboratorio de Virus Respiratorio | Centers for Disease Control and Prevention |
| EPI353408 | HA (H1) | Mexico | 2011-Dec-28 | A/Mexico/52/2011 | Laboratorio de Virus Respiratorio | Centers for Disease Control and Prevention |
| EPI320045 | HA (H1) | Mexico | 2011-Feb-22 | A/Mexico/1658/2011 | Laboratorio de Virus Respiratorio | Centers for Disease Control and Prevention |
| EPI331210 | HA (H1) | Mexico | 2011-Mar-15 | A/Mexico/2208/2011 | Laboratorio de Virus Respiratorio | Centers for Disease Control and Prevention |
| EPI330992 | HA (H1) | Mexico | 2011-Mar-22 | A/Mexico/1946/2011 | Laboratorio de Virus Respiratorio | Centers for Disease Control and Prevention |
| EPI391298 | HA (H1) | Mexico | 2012-Feb-13 | A/Mexico/5698/2012 | Laboratorio de Virus Respiratorio | Centers for Disease Control and Prevention |
| EPI353402 | HA (H1) | Mexico | 2012-Jan-08 | A/Mexico/210/2012 | Laboratorio de Virus Respiratorio | Centers for Disease Control and Prevention |
| EPI508443 | HA (H1) | Mexico | 2013-Aug-31 | A/Mexico/2733/2013 | Laboratorio de Virus Respiratorio | Centers for Disease Control and Prevention |
| EPI504791 | HA (H1) | Mexico | 2013-Dec-27 | A/Mexico/06/2013 | Laboratorio de Virus Respiratorio | Centers for Disease Control and Prevention |
| EPI467233 | HA (H1) | Mexico | 2013-Jul-13 | A/Mexico/2410/2013 | Laboratorio de Virus Respiratorio | Centers for Disease Control and Prevention |
| EPI509496 | HA (H1) | Mexico | 2013-Mar-01 | A/Mexico/01/2013 | Laboratorio de Virus Respiratorio | Centers for Disease Control and Prevention |
| EPI503827 | HA (H1) | Mexico | 2013-Oct-21 | A/Mexico/3280/2013 | Laboratorio de Virus Respiratorio | Centers for Disease Control and Prevention |
| EPI183311 | HA (H1) | Netherlands | 2009-Jan-01 | A/Netherlands/602/2009 | Erasmus University of Rotterdam | Centers for Disease Control and Prevention |
| EPI249092 | HA (H1) | Nicaragua | 2009-Aug-15 | A/Managua/3275.01/2009 |  | Other Database Import |
| EPI249036 | HA (H1) | Nicaragua | 2009-Aug-18 | A/Managua/164.01/2009 |  | Other Database Import |
| EPI249582 | HA (H1) | Nicaragua | 2009-Aug-18 | A/Managua/2323.02/2009 |  | Other Database Import |
| EPI227690 | HA (H1) | Nicaragua | 2009-Aug-25 | A/Managua/6502/2009 | Laboratorio de Virologia, Direccion de Microbiologia | Centers for Disease Control and Prevention |
| EPI275081 | HA (H1) | Nicaragua | 2009-Jul-21 | A/Managua/5364.01/2009 |  | Other Database Import |
| EPI227684 | HA (H1) | Nicaragua | 2009-Jul-27 | A/Managua/46601/2009 | Laboratorio de Virologia, Direccion de Microbiologia | Centers for Disease Control and Prevention |
| EPI237350 | HA (H1) | Nicaragua | 2009-Jun-01 | A/Managua/4467.05/2009 |  | Other Database Import |
| EPI249068 | HA (H1) | Nicaragua | 2009-Jun-16 | A/Managua/2330.02/2009 |  | Other Database Import |
| EPI190855 | HA (H1) | Nicaragua | 2009-Jun-26 | A/Managua/0536N/2009 |  | Other Database Import |
| EPI273844 | HA (H1) | Nicaragua | 2010-Apr-01 | A/Nicaragua/322/2010 | Laboratorio de Virologia, Direccion de Microbiologia | Centers for Disease Control and Prevention |
| EPI346507 | HA (H1) | Nicaragua | 2011-Oct-10 | A/Managua/748/2011 | Laboratorio de Virologia, Direccion de Microbiologia | Centers for Disease Control and Prevention |
| EPI465282 | HA (H1) | Nicaragua | 2013-Apr-14 | A/Managua/601/2013 | Laboratorio de Virologia, Direccion de Microbiologia | Centers for Disease Control and Prevention |
| EPI465279 | HA (H1) | Nicaragua | 2013-Apr-20 | A/Managua/687/2013 | Laboratorio de Virologia, Direccion de Microbiologia | Centers for Disease Control and Prevention |
| EPI465462 | HA (H1) | Nicaragua | 2013-Jun-14 | A/Managua/30074.01/2013 | Laboratorio de Virologia, Direccion de Microbiologia | Centers for Disease Control and Prevention |
| EPI211485 | HA (H1) | Panama | 2009-Jun-18 | A/Panama/4252/2009 | Instituto Conmemorativo Gorgas de Estudios de la Salud | Centers for Disease Control and Prevention |
| EPI183241 | HA (H1) | Panama | 2009-May-11 | A/Panama/302869/2009 | Instituto Conmemorativo Gorgas de Estudios de la Salud | Centers for Disease Control and Prevention |
| EPI278852 | HA (H1) | Panama | 2010-Jun-07 | A/Panama/307168/2010 | Instituto Conmemorativo Gorgas de Estudios de la Salud | Centers for Disease Control and Prevention |
| EPI278854 | HA (H1) | Panama | 2010-Jun-14 | A/Panama/307207/2010 | Instituto Conmemorativo Gorgas de Estudios de la Salud | Centers for Disease Control and Prevention |
| EPI335822 | HA (H1) | Panama | 2011-Jun-10 | A/Panama/309335/2011 | Instituto Conmemorativo Gorgas de Estudios de la Salud | Centers for Disease Control and Prevention |
| EPI394749 | HA (H1) | Panama | 2012-Jun-05 | A/Panama/310521/2012 | Instituto Conmemorativo Gorgas de Estudios de la Salud | Centers for Disease Control and Prevention |
| EPI468142 | HA (H1) | Panama | 2013-Jun-13 | A/Panama/313106/2013 | Instituto Conmemorativo Gorgas de Estudios de la Salud | Centers for Disease Control and Prevention |
| EPI467381 | HA (H1) | Panama | 2013-Jun-25 | A/Panama/313202/2013 | Instituto Conmemorativo Gorgas de Estudios de la Salud | Centers for Disease Control and Prevention |
| EPI217082 | HA (H1) | Puerto Rico | 2009-Aug-07 | A/Puerto Rico/50/2009 | Puerto Rico Department of Health | Centers for Disease Control and Prevention |
| EPI240403 | HA (H1) | Puerto Rico | 2009-Dec-06 | A/Puerto Rico/51/2009 | Puerto Rico Department of Health | Centers for Disease Control and Prevention |
| EPI273751 | HA (H1) | Puerto Rico | 2009-Dec-18 | A/Puerto Rico/20/2009 | Puerto Rico Department of Health | Centers for Disease Control and Prevention |
| EPI346510 | HA (H1) | Puerto Rico | 2011-Aug-03 | A/Puerto Rico/04/2011 | Puerto Rico Department of Health | Centers for Disease Control and Prevention |
| EPI366314 | HA (H1) | Puerto Rico | 2011-Aug-18 | A/Puerto Rico/25/2011 | Puerto Rico Department of Health | Centers for Disease Control and Prevention |
| EPI316427 | HA (H1) | Puerto Rico | 2011-Feb-04 | A/Puerto Rico/01/2011 | Puerto Rico Department of Health | Centers for Disease Control and Prevention |
| EPI341961 | HA (H1) | Puerto Rico | 2011-Jun-24 | A/Puerto Rico/02/2011 | Puerto Rico Department of Health | Centers for Disease Control and Prevention |
| EPI366308 | HA (H1) | Puerto Rico | 2011-May-31 | A/Puerto Rico/05/2011 | Puerto Rico Department of Health | Centers for Disease Control and Prevention |
| EPI349362 | HA (H1) | Puerto Rico | 2011-Nov-21 | A/Puerto Rico/8233/2011 | Puerto Rico Department of Health | Centers for Disease Control and Prevention |
| EPI347556 | HA (H1) | Puerto Rico | 2011-Oct-22 | A/Puerto Rico/21/2011 | Puerto Rico Department of Health | Centers for Disease Control and Prevention |
| EPI368690 | HA (H1) | Puerto Rico | 2012-Feb-14 | A/Puerto Rico/01/2012 | Puerto Rico Department of Health | Centers for Disease Control and Prevention |
| EPI397629 | HA (H1) | Puerto Rico | 2012-Jul-03 | A/Puerto Rico/43/2012 | Puerto Rico Department of Health | Centers for Disease Control and Prevention |
| EPI391293 | HA (H1) | Puerto Rico | 2012-Jun-07 | A/Puerto Rico/06/2012 | Puerto Rico Department of Health | Centers for Disease Control and Prevention |
| EPI396142 | HA (H1) | Puerto Rico | 2012-Jun-29 | A/Puerto Rico/39/2012 | Puerto Rico Department of Health | Centers for Disease Control and Prevention |
| EPI391282 | HA (H1) | Puerto Rico | 2012-May-18 | A/Puerto Rico/05/2012 | Puerto Rico Department of Health | Centers for Disease Control and Prevention |
| EPI509486 | HA (H1) | Puerto Rico | 2013-Dec-16 | A/Puerto Rico/21/2013 | Puerto Rico Department of Health | Centers for Disease Control and Prevention |
| EPI465070 | HA (H1) | Puerto Rico | 2013-Jun-14 | A/Puerto Rico/01/2013 | Puerto Rico Department of Health | Centers for Disease Control and Prevention |
| EPI465269 | HA (H1) | Puerto Rico | 2013-Jun-14 | A/Puerto Rico/05/2013 | Puerto Rico Department of Health | Centers for Disease Control and Prevention |
| EPI486392 | HA (H1) | Puerto Rico | 2013-Oct-01 | A/Puerto Rico/18/2013 | Puerto Rico Department of Health | Centers for Disease Control and Prevention |
| EPI217088 | HA (H1) | Saint Kitts and Nevis, Federation of | 2009-Jul-29 | A/Saint Kitts/7274/2009 | Caribbean Epidemiology Center | Centers for Disease Control and Prevention |
| EPI217091 | HA (H1) | Saint Lucia | 2009-Jul-31 | A/Saint Lucia/7178/2009 | Caribbean Epidemiology Center | Centers for Disease Control and Prevention |
| EPI231341 | HA (H1) | Saint Lucia | 2009-Sep-25 | A/St. Lucia/9333/2009 | Caribbean Epidemiology Center | Centers for Disease Control and Prevention |
| EPI497714 | HA (H1) | Saint Vincent and the Grenadines | 2013-Oct-08 | A/St. Vincent and Grenadines/3292/2013 | Caribbean Epidemiology Center | Centers for Disease Control and Prevention |
| EPI186300 | HA (H1) | Trinidad and Tobago | 2009-Jan-01 | A/Trinidad/4601/2009 |  | Centers for Disease Control and Prevention |
| EPI231350 | HA (H1) | Trinidad and Tobago | 2009-Oct-22 | A/Trinidad/9184/2009 | Caribbean Epidemiology Center | Centers for Disease Control and Prevention |
| EPI335837 | HA (H1) | Trinidad and Tobago | 2011-Jul-08 | A/Trinidad/1648/2011 | Caribbean Epidemiology Center | Centers for Disease Control and Prevention |
| EPI460142 | HA (H1) | Trinidad and Tobago | 2013-Mar-01 | A/Trinidad/979/2013 | Caribbean Epidemiology Center | Centers for Disease Control and Prevention |
| EPI459774 | HA (H1) | Trinidad and Tobago | 2013-Mar-13 | A/Trinidad/982/2013 | Caribbean Epidemiology Center | Centers for Disease Control and Prevention |
| EPI497649 | HA (H1) | Trinidad and Tobago | 2013-Oct-24 | A/Trinidad/3568/2013 | Caribbean Epidemiology Center | Centers for Disease Control and Prevention |
| EPI231353 | HA (H1) | Turks and Caicos Islands | 2009-Oct-13 | A/Turks And Caicos/9060/2009 | Caribbean Epidemiology Center | Centers for Disease Control and Prevention |
| EPI320155 | HA (H1) | Turks and Caicos Islands | 2011-Jan-25 | A/Turks and Caicos/211/2011 | Caribbean Epidemiology Center | Centers for Disease Control and Prevention |
| EPI516535 | HA (H1) | United States | 2009-Apr-09 | A/California/07/2009 | Naval Health Research Center | Centers for Disease Control and Prevention |
| EPI341574 | HA (H1) | United States | 2009-Jan-01 | A/California/04/2009 |  | Other Database Import |
| EPI326251 | HA (H3) | Anguilla | 2011-Jan-24 | A/Anguilla/273/2011 | Caribbean Epidemiology Center | Centers for Disease Control and Prevention |
| EPI459802 | HA (H3) | Anguilla | 2013-Jan-10 | A/Anguilla/104/2013 | Caribbean Epidemiology Center | Centers for Disease Control and Prevention |
| EPI459802 | HA (H3) | Anguilla | 2013-Jan-10 | A/Anguilla/104/2013 | Caribbean Epidemiology Center | Centers for Disease Control and Prevention |
| EPI590418 | HA (H3) | Aruba | 2014-Dec-12 | A/Netherlands/781/2014 | National Institute for Public Health and the Environment (RIVM) | National Institute for Public Health and the Environment (RIVM) |
| EPI176959 | HA (H3) | Australia | 2007-Jan-01 | A/Brisbane/10/2007 |  | Other Database Import |
| EPI211334 | HA (H3) | Australia | 2009-Jan-01 | A/Perth/16/2009 | WHO Collaborating Centre for Reference and Research on Influenza | Centers for Disease Control and Prevention |
| EPI513286 | HA (H3) | Australia | 2011-Oct-24 | A/Victoria/361/2011 | WHO Collaborating Centre for Reference and Research on Influenza | Centers for Disease Control and Prevention |
| EPI394812 | HA (H3) | Bermuda | 2012-Jun-13 | A/Bermuda/1915/2012 | Caribbean Epidemiology Center | Centers for Disease Control and Prevention |
| EPI232523 | HA (H3) | Cayman Islands | 2009-Oct-22 | A/Cayman Islands/9266/2009 | Caribbean Epidemiology Center | Centers for Disease Control and Prevention |
| EPI155501 | HA (H3) | Costa Rica | 2006-Nov-09 | A/Costa Rica/7322/2006 |  | Centers for Disease Control and Prevention |
| EPI155503 | HA (H3) | Costa Rica | 2007-Jan-05 | A/Costa Rica/176/2007 |  | Centers for Disease Control and Prevention |
| EPI157551 | HA (H3) | Costa Rica | 2007-May-25 | A/Costa Rica/4082/2007 |  | Centers for Disease Control and Prevention |
| EPI161990 | HA (H3) | Costa Rica | 2007-May-25 | A/Costa Rica/4082/2007 |  | Centers for Disease Control and Prevention |
| EPI172432 | HA (H3) | Costa Rica | 2008-Jun-19 | A/Costa Rica/7172/2008 |  | Centers for Disease Control and Prevention |
| EPI211317 | HA (H3) | Costa Rica | 2009-Jun-05 | A/Costa Rica/5179/2009 | Laboratorio Nacional de Influenza | Centers for Disease Control and Prevention |
| EPI349761 | HA (H3) | Costa Rica | 2011-Nov-11 | A/Costa Rica/8211/2011 | Laboratorio Nacional de Influenza | Centers for Disease Control and Prevention |
| EPI397057 | HA (H3) | Costa Rica | 2012-Jul-17 | A/Costa Rica/9093/2012 | Laboratorio Nacional de Influenza | Centers for Disease Control and Prevention |
| EPI484551 | HA (H3) | Costa Rica | 2013-Jul-23 | A/Costa Rica/4009/2013 | Laboratorio Nacional de Influenza | Centers for Disease Control and Prevention |
| EPI484551 | HA (H3) | Costa Rica | 2013-Jul-23 | A/Costa Rica/4009/2013 | Laboratorio Nacional de Influenza | Centers for Disease Control and Prevention |
| EPI547806 | HA (H3) | Costa Rica | 2014-Jul-09 | A/Costa Rica/2303/2014 | Laboratorio Nacional de Influenza | Centers for Disease Control and Prevention |
| EPI155856 | HA (H3) | Dominica | 2007-Feb-25 | A/Dominican Republic/2896/2007 |  | Centers for Disease Control and Prevention |
| EPI459805 | HA (H3) | Dominica | 2013-Jan-21 | A/Dominica/653/2013 | Caribbean Epidemiology Center | Centers for Disease Control and Prevention |
| EPI459805 | HA (H3) | Dominica | 2013-Jan-21 | A/Dominica/653/2013 | Caribbean Epidemiology Center | Centers for Disease Control and Prevention |
| EPI254592 | HA (H3) | Dominican Republic | 2009-Dec-04 | A/Dominican Republic/3743/2009 | Laboratorio de Investigacion / Centro de Educacion Medica y Amistad Dominico Japones (CEMADOJA) | Centers for Disease Control and Prevention |
| EPI211266 | HA (H3) | Dominican Republic | 2009-May-25 | A/Dominican Republic/988/2009 | Laboratorio de Investigacion / Centro de Educacion Medica y Amistad Dominico Japones (CEMADOJA) | Centers for Disease Control and Prevention |
| EPI279996 | HA (H3) | Dominican Republic | 2010-Aug-03 | A/Dominican Republic/4389/2010 | Laboratorio de Investigacion / Centro de Educacion Medica y Amistad Dominico Japones (CEMADOJA) | Centers for Disease Control and Prevention |
| EPI325849 | HA (H3) | Dominican Republic | 2010-Jun-29 | A/Santo Domingo/WRAIR3514T/2010 |  | Other Database Import |
| EPI395233 | HA (H3) | Dominican Republic | 2012-Jun-04 | A/Dominican Republic/6524/2012 | Laboratorio de Investigacion / Centro de Educacion Medica y Amistad Dominico Japones (CEMADOJA) | Centers for Disease Control and Prevention |
| EPI547821 | HA (H3) | Dominican Republic | 2014-May-22 | A/Dominican Republic/8490/2014 | Laboratorio de Investigacion / Centro de Educacion Medica y Amistad Dominico Japones (CEMADOJA) | Centers for Disease Control and Prevention |
| EPI547814 | HA (H3) | Dominican Republic | 2014-May-28 | A/Dominican Republic/8483/2014 | Laboratorio de Investigacion / Centro de Educacion Medica y Amistad Dominico Japones (CEMADOJA) | Centers for Disease Control and Prevention |
| EPI157579 | HA (H3) | El Salvador | 2007-Sep-03 | A/El Salvador/579/2007 |  | Centers for Disease Control and Prevention |
| EPI161999 | HA (H3) | El Salvador | 2007-Sep-03 | A/El Salvador/579/2007 |  | Centers for Disease Control and Prevention |
| EPI279985 | HA (H3) | El Salvador | 2010-Aug-06 | A/El Salvador/1060/2010 | Contiguo a Hospital Rosales | Centers for Disease Control and Prevention |
| EPI295104 | HA (H3) | El Salvador | 2010-Aug-31 | A/El Salvador/1103/2010 | Contiguo a Hospital Rosales | Centers for Disease Control and Prevention |
| EPI278812 | HA (H3) | El Salvador | 2010-Jun-08 | A/El Salvador/636/2010 | Contiguo a Hospital Rosales | Centers for Disease Control and Prevention |
| EPI346446 | HA (H3) | El Salvador | 2011-Oct-03 | A/El Salvador/1571/2011 | Contiguo a Hospital Rosales | Centers for Disease Control and Prevention |
| EPI347486 | HA (H3) | El Salvador | 2011-Sep-21 | A/El Salvador/1513/2011 | Contiguo a Hospital Rosales | Centers for Disease Control and Prevention |
| EPI459841 | HA (H3) | El Salvador | 2013-Mar-18 | A/El Salvador/433/2013 | Contiguo a Hospital Rosales | Centers for Disease Control and Prevention |
| EPI459841 | HA (H3) | El Salvador | 2013-Mar-18 | A/El Salvador/433/2013 | Contiguo a Hospital Rosales | Centers for Disease Control and Prevention |
| EPI309767 | HA (H3) | Guadeloupe | 2010-Nov-30 | A/GUADELOUPE/202/2010 | Centers for Disease Control and Prevention | WHO Collaborating Centre for Reference and Research on Influenza |
| EPI342275 | HA (H3) | Guadeloupe | 2010-Nov-30 | A/Guadeloupe/201/2010 | National Influenza Center French Guiana and French Indies | Centers for Disease Control and Prevention |
| EPI531738 | HA (H3) | Guadeloupe | 2014-Feb-10 | A/Guadeloupe/4127/2014 | National Influenza Center French Guiana and French Indies | Centers for Disease Control and Prevention |
| EPI163130 | HA (H3) | Guatemala | 2008-May-19 | A/Guatemala/494/2008 |  | Centers for Disease Control and Prevention |
| EPI193980 | HA (H3) | Guatemala | 2009-Jul-09 | A/Guatemala/29/2009 | Laboratorio Nacional De Salud Guatemala | Centers for Disease Control and Prevention |
| EPI193924 | HA (H3) | Guatemala | 2009-Jun-03 | A/Guatemala/1066/2009 | Laboratorio Nacional De Salud Guatemala | Centers for Disease Control and Prevention |
| EPI211337 | HA (H3) | Guatemala | 2009-Jun-15 | A/Guatemala/1913/2009 | Laboratorio Nacional De Salud Guatemala | Centers for Disease Control and Prevention |
| EPI342193 | HA (H3) | Guatemala | 2010-Jul-13 | A/Guatemala/591/2010 | Laboratorio Nacional De Salud Guatemala | Centers for Disease Control and Prevention |
| EPI301071 | HA (H3) | Guatemala | 2010-Oct-05 | A/Guatemala/754/2010 | Laboratorio Nacional De Salud Guatemala | Centers for Disease Control and Prevention |
| EPI295095 | HA (H3) | Guatemala | 2010-Sep-02 | A/Guatemala/690/2010 | Laboratorio Nacional De Salud Guatemala | Centers for Disease Control and Prevention |
| EPI326291 | HA (H3) | Guatemala | 2011-Mar-02 | A/Guatemala/51/2011 | Laboratorio Nacional De Salud Guatemala | Centers for Disease Control and Prevention |
| EPI326288 | HA (H3) | Guatemala | 2011-May-25 | A/Guatemala/199/2011 | Laboratorio Nacional De Salud Guatemala | Centers for Disease Control and Prevention |
| EPI340921 | HA (H3) | Guatemala | 2011-Sep-02 | A/Guatemala/453/2011 | Laboratorio Nacional De Salud Guatemala | Centers for Disease Control and Prevention |
| EPI468141 | HA (H3) | Guatemala | 2013-Jul-09 | A/Guatemala/287/2013 | Laboratorio Nacional De Salud Guatemala | Centers for Disease Control and Prevention |
| EPI468141 | HA (H3) | Guatemala | 2013-Jul-09 | A/Guatemala/287/2013 | Laboratorio Nacional De Salud Guatemala | Centers for Disease Control and Prevention |
| EPI541604 | HA (H3) | Guatemala | 2014-May-07 | A/Guatemala/3550/2014 | Laboratorio Nacional De Salud Guatemala | Centers for Disease Control and Prevention |
| EPI566009 | HA (H3) | Guatemala | 2014-Nov-04 | A/Guatemala/179/2014 | Laboratorio Nacional De Salud Guatemala | Centers for Disease Control and Prevention |
| EPI564325 | HA (H3) | Guatemala | 2014-Nov-12 | A/Guatemala/4638/2014 | Laboratorio Nacional De Salud Guatemala | Centers for Disease Control and Prevention |
| EPI564330 | HA (H3) | Guatemala | 2014-Nov-13 | A/Guatemala/5211/2014 | Laboratorio Nacional De Salud Guatemala | Centers for Disease Control and Prevention |
| EPI565405 | HA (H3) | Guatemala | 2014-Nov-14 | A/Guatemala/4647/2014 | Laboratorio Nacional De Salud Guatemala | Centers for Disease Control and Prevention |
| EPI565686 | HA (H3) | Guatemala | 2014-Oct-26 | A/Guatemala/171/2014 | Laboratorio Nacional De Salud Guatemala | Centers for Disease Control and Prevention |
| EPI232463 | HA (H3) | Haiti | 2009-Jul-01 | A/Haiti/66/2009 | Laboratoire National de Sante Publique | Centers for Disease Control and Prevention |
| EPI155203 | HA (H3) | Honduras | 2006-Oct-01 | A/Honduras/1922/2006 |  | Centers for Disease Control and Prevention |
| EPI157664 | HA (H3) | Honduras | 2007-Oct-05 | A/Honduras/6374/2007 |  | Centers for Disease Control and Prevention |
| EPI162066 | HA (H3) | Honduras | 2007-Oct-05 | A/Honduras/6374/2007 |  | Centers for Disease Control and Prevention |
| EPI157670 | HA (H3) | Honduras | 2007-Oct-11 | A/Honduras/6453/2007 |  | Centers for Disease Control and Prevention |
| EPI162072 | HA (H3) | Honduras | 2007-Oct-11 | A/Honduras/6453/2007 |  | Centers for Disease Control and Prevention |
| EPI193974 | HA (H3) | Honduras | 2009-Jun-30 | A/Honduras/2243/2009 | Laboratorio Nacional de Virologia | Centers for Disease Control and Prevention |
| EPI185779 | HA (H3) | Honduras | 2009-May-05 | A/Honduras/56/2009 | Laboratorio Nacional de Virologia | Centers for Disease Control and Prevention |
| EPI185785 | HA (H3) | Jamaica | 2009-Apr-28 | A/Jamaica/2970/2009 | Caribbean Epidemiology Center | Centers for Disease Control and Prevention |
| EPI157956 | HA (H3) | Martinique | 2007-Feb-09 | A/Martinique/12/2007 |  | Centers for Disease Control and Prevention |
| EPI162123 | HA (H3) | Martinique | 2007-Feb-09 | A/Martinique/12/2007 |  | Centers for Disease Control and Prevention |
| EPI157966 | HA (H3) | Mexico | 2007-Oct-18 | A/Mexico/3701/2007 |  | Centers for Disease Control and Prevention |
| EPI162124 | HA (H3) | Mexico | 2007-Oct-18 | A/Mexico/3701/2007 |  | Centers for Disease Control and Prevention |
| EPI463946 | HA (H3) | Mexico | 2008-Dec-01 | A/Mexico/24013/2008 |  | Other Database Import |
| EPI463954 | HA (H3) | Mexico | 2008-Dec-01 | A/Mexico/24014/2008 |  | Other Database Import |
| EPI463938 | HA (H3) | Mexico | 2008-Dec-02 | A/Mexico/24012/2008 |  | Other Database Import |
| EPI336112 | HA (H3) | Mexico | 2008-Jan-10 | A/Mexico/UASLP-011/2008 |  | Other Database Import |
| EPI336107 | HA (H3) | Mexico | 2008-Jan-14 | A/Mexico/UASLP-012/2008 |  | Other Database Import |
| EPI162130 | HA (H3) | Mexico | 2008-Jan-17 | A/Mexico/499/2008 |  | Centers for Disease Control and Prevention |
| EPI463833 | HA (H3) | Mexico | 2008-Nov-01 | A/Mexico/24003/2008 |  | Other Database Import |
| EPI463901 | HA (H3) | Mexico | 2008-Nov-01 | A/Mexico/24009/2008 |  | Other Database Import |
| EPI463917 | HA (H3) | Mexico | 2008-Nov-01 | A/Mexico/24011/2008 |  | Other Database Import |
| EPI463887 | HA (H3) | Mexico | 2009-Apr-01 | A/Mexico/24008/2009 |  | Other Database Import |
| EPI243572 | HA (H3) | Mexico | 2009-Aug-07 | A/Mexico/5216/2009 | Laboratorio de Virus Respiratorio | Centers for Disease Control and Prevention |
| EPI301119 | HA (H3) | Mexico | 2010-Aug-13 | A/Mexico/4268/2010 | Laboratorio de Virus Respiratorio | Centers for Disease Control and Prevention |
| EPI295208 | HA (H3) | Mexico | 2010-Aug-27 | A/Mexico/4391/2010 | Laboratorio de Virus Respiratorio | Centers for Disease Control and Prevention |
| EPI295227 | HA (H3) | Mexico | 2010-Aug-31 | A/Mexico/4674/2010 | Laboratorio de Virus Respiratorio | Centers for Disease Control and Prevention |
| EPI325913 | HA (H3) | Mexico | 2010-Dec-22 | A/Mexico City/WRAIR3570T/2010 |  | Other Database Import |
| EPI325921 | HA (H3) | Mexico | 2010-Dec-22 | A/Mexico City/WRAIR3570T/2010 |  | Other Database Import |
| EPI325801 | HA (H3) | Mexico | 2010-Jan-26 | A/Mexico City/WRAIR1752T/2010 |  | Other Database Import |
| EPI309389 | HA (H3) | Mexico | 2010-Nov-04 | A/Mexico/6998/2010 | Laboratorio de Virus Respiratorio | Centers for Disease Control and Prevention |
| EPI331240 | HA (H3) | Mexico | 2011-Apr-08 | A/Mexico/2554/2011 | Laboratorio de Virus Respiratorio | Centers for Disease Control and Prevention |
| EPI565836 | HA (H3) | Mexico | 2014-Dec-01 | A/Mexico/2865/2014 | Laboratorio de Virus Respiratorio | Centers for Disease Control and Prevention |
| EPI565804 | HA (H3) | Mexico | 2014-Dec-02 | A/Mexico/836/2014 | Laboratorio de Virus Respiratorio | Centers for Disease Control and Prevention |
| EPI560582 | HA (H3) | Mexico | 2014-Dec-04 | A/Mexico/2971/2014 | Laboratorio de Virus Respiratorio | Centers for Disease Control and Prevention |
| EPI560615 | HA (H3) | Mexico | 2014-Dec-08 | A/Mexico/2974/2014 | Laboratorio de Virus Respiratorio | Centers for Disease Control and Prevention |
| EPI565844 | HA (H3) | Mexico | 2014-Dec-09 | A/Mexico/3005/2014 | Laboratorio de Virus Respiratorio | Centers for Disease Control and Prevention |
| EPI565849 | HA (H3) | Mexico | 2014-Dec-17 | A/Mexico/3040/2014 | Laboratorio de Virus Respiratorio | Centers for Disease Control and Prevention |
| EPI547917 | HA (H3) | Mexico | 2014-Feb-12 | A/Mexico/711/2014 | Laboratorio de Virus Respiratorio | Centers for Disease Control and Prevention |
| EPI565372 | HA (H3) | Mexico | 2014-Nov-14 | A/Mexico/2720/2014 | Laboratorio de Virus Respiratorio | Centers for Disease Control and Prevention |
| EPI565699 | HA (H3) | Mexico | 2014-Nov-21 | A/Mexico/2835/2014 | Laboratorio de Virus Respiratorio | Centers for Disease Control and Prevention |
| EPI565385 | HA (H3) | Mexico | 2014-Nov-24 | A/Mexico/2777/2014 | Laboratorio de Virus Respiratorio | Centers for Disease Control and Prevention |
| EPI565365 | HA (H3) | Mexico | 2014-Nov-26 | A/Mexico/2789/2014 | Laboratorio de Virus Respiratorio | Centers for Disease Control and Prevention |
| EPI173816 | HA (H3) | Nicaragua | 2007-Aug-03 | A/Managua/4902.01/2007 |  | Other Database Import |
| EPI162156 | HA (H3) | Nicaragua | 2007-Jan-01 | A/Managua/2760.01/2007 |  | Centers for Disease Control and Prevention |
| EPI178230 | HA (H3) | Nicaragua | 2007-Jun-05 | A/Managua/4348.01/2007 |  | Other Database Import |
| EPI154122 | HA (H3) | Nicaragua | 2007-Jun-12 | A/Managua/33/2007 |  | Other Database Import |
| EPI154034 | HA (H3) | Nicaragua | 2007-Jun-22 | A/Managua/20/2007 |  | Other Database Import |
| EPI154074 | HA (H3) | Nicaragua | 2007-Jun-22 | A/Managua/29/2007 |  | Other Database Import |
| EPI154098 | HA (H3) | Nicaragua | 2007-Jun-23 | A/Managua/34/2007 |  | Other Database Import |
| EPI154444 | HA (H3) | Nicaragua | 2007-Jun-27 | A/Managua/25/2007 |  | Other Database Import |
| EPI315224 | HA (H3) | Nicaragua | 2010-Jun-01 | A/Managua/1155.01/2010 |  | Other Database Import |
| EPI315772 | HA (H3) | Nicaragua | 2010-Jun-07 | A/Managua/3209.01/2010 |  | Other Database Import |
| EPI277421 | HA (H3) | Nicaragua | 2010-Jun-10 | A/Managua/2867.01/2010 |  | Other Database Import |
| EPI396164 | HA (H3) | Nicaragua | 2012-Jul-10 | A/Managua/726.03/2012 | Laboratorio de Virologia, Direccion de Microbiologia | Centers for Disease Control and Prevention |
| EPI465947 | HA (H3) | Nicaragua | 2013-May-07 | A/Esteli/816/2013 | Laboratorio de Virologia, Direccion de Microbiologia | Centers for Disease Control and Prevention |
| EPI465947 | HA (H3) | Nicaragua | 2013-May-07 | A/Esteli/816/2013 | Laboratorio de Virologia, Direccion de Microbiologia | Centers for Disease Control and Prevention |
| EPI548001 | HA (H3) | Nicaragua | 2014-Jun-25 | A/Nicaragua/7402/2014 | Laboratorio de Virologia, Direccion de Microbiologia | Centers for Disease Control and Prevention |
| EPI185802 | HA (H3) | Puerto Rico | 2009-Feb-09 | A/Puerto Rico/18/2009 | Puerto Rico Department of Health | Centers for Disease Control and Prevention |
| EPI193918 | HA (H3) | Puerto Rico | 2009-Jun-02 | A/Puerto Rico/46/2009 | Puerto Rico Department of Health | Centers for Disease Control and Prevention |
| EPI193906 | HA (H3) | Puerto Rico | 2009-May-26 | A/Puerto Rico/25/2009 | Puerto Rico Department of Health | Centers for Disease Control and Prevention |
| EPI295256 | HA (H3) | Puerto Rico | 2010-Sep-24 | A/Puerto Rico/01/2010 | Puerto Rico Department of Health | Centers for Disease Control and Prevention |
| EPI378216 | HA (H3) | Puerto Rico | 2011-Dec-07 | A/Puerto Rico/38/2011 | Centers for Disease Control and Prevention | Centers for Disease Control and Prevention |
| EPI377477 | HA (H3) | Puerto Rico | 2011-Nov-20 | A/Puerto Rico/36/2011 | Centers for Disease Control and Prevention | Centers for Disease Control and Prevention |
| EPI353489 | HA (H3) | Puerto Rico | 2011-Nov-26 | A/Puerto Rico/30/2011 | Puerto Rico Department of Health | Centers for Disease Control and Prevention |
| EPI347536 | HA (H3) | Puerto Rico | 2011-Oct-20 | A/Puerto Rico/23/2011 | Puerto Rico Department of Health | Centers for Disease Control and Prevention |
| EPI371811 | HA (H3) | Puerto Rico | 2012-Feb-12 | A/Puerto Rico/03/2012 | Puerto Rico Department of Health | Centers for Disease Control and Prevention |
| EPI387763 | HA (H3) | Puerto Rico | 2012-Jun-03 | A/Puerto Rico/04/2012 | Puerto Rico Department of Health | Centers for Disease Control and Prevention |
| EPI391271 | HA (H3) | Puerto Rico | 2012-Jun-11 | A/Puerto Rico/08/2012 | Puerto Rico Department of Health | Centers for Disease Control and Prevention |
| EPI394787 | HA (H3) | Puerto Rico | 2012-Jun-14 | A/Puerto Rico/23/2012 | Puerto Rico Department of Health | Centers for Disease Control and Prevention |
| EPI394790 | HA (H3) | Puerto Rico | 2012-Jun-26 | A/Puerto Rico/34/2012 | Puerto Rico Department of Health | Centers for Disease Control and Prevention |
| EPI397054 | HA (H3) | Puerto Rico | 2012-Jun-27 | A/Puerto Rico/36/2012 | Puerto Rico Department of Health | Centers for Disease Control and Prevention |
| EPI371957 | HA (H3) | Puerto Rico | 2012-Mar-02 | A/Puerto Rico/02/2012 | Puerto Rico Department of Health | Centers for Disease Control and Prevention |
| EPI537872 | HA (H3) | Puerto Rico | 2014-Apr-14 | A/Puerto Rico/14/2014 | Puerto Rico Department of Health | Centers for Disease Control and Prevention |
| EPI540027 | HA (H3) | Puerto Rico | 2014-Jun-16 | A/Puerto Rico/23/2014 | Puerto Rico Department of Health | Centers for Disease Control and Prevention |
| EPI540068 | HA (H3) | Puerto Rico | 2014-Jun-18 | A/Puerto Rico/19/2014 | Puerto Rico Department of Health | Centers for Disease Control and Prevention |
| EPI540034 | HA (H3) | Puerto Rico | 2014-Jun-19 | A/Puerto Rico/22/2014 | Puerto Rico Department of Health | Centers for Disease Control and Prevention |
| EPI571982 | HA (H3) | Puerto Rico | 2014-Nov-03 | A/Puerto Rico/27/2014 | Puerto Rico Department of Health | Centers for Disease Control and Prevention |
| EPI572184 | HA (H3) | Puerto Rico | 2014-Nov-04 | A/Puerto Rico/28/2014 | Puerto Rico Department of Health | Centers for Disease Control and Prevention |
| EPI185949 | HA (H3) | Trinidad and Tobago | 2009-Jan-01 | A/Trinidad/2940/2009 |  | Centers for Disease Control and Prevention |
| EPI459823 | HA (H3) | Trinidad and Tobago | 2013-Mar-19 | A/Trinidad/1117/2013 | Caribbean Epidemiology Center | Centers for Disease Control and Prevention |
| EPI459823 | HA (H3) | Trinidad and Tobago | 2013-Mar-19 | A/Trinidad/1117/2013 | Caribbean Epidemiology Center | Centers for Disease Control and Prevention |
| EPI497653 | HA (H3) | Trinidad and Tobago | 2013-Oct-27 | A/Trinidad/3558/2013 | Caribbean Epidemiology Center | Centers for Disease Control and Prevention |
| EPI497653 | HA (H3) | Trinidad and Tobago | 2013-Oct-27 | A/Trinidad/3558/2013 | Caribbean Epidemiology Center | Centers for Disease Control and Prevention |
| EPI538476 | HA (H3) | Trinidad and Tobago | 2014-Feb-28 | A/Trinidad/753/2014 | Caribbean Epidemiology Center | Centers for Disease Control and Prevention |
| EPI537015 | HA (H3) | United States | 2012-Apr-15 | A/Texas/50/2012 | Texas Department of State Health Services-Laboratory Services | Centers for Disease Control and Prevention |
| EPI216723 | NA (N1) | Barbados | 2009-Sep-04 | A/Barbados/275/2009 | Public Health Laboratory | Centers for Disease Control and Prevention |
| EPI216726 | NA (N1) | Barbados | 2009-Sep-10 | A/Barbados/280/2009 | Public Health Laboratory | Centers for Disease Control and Prevention |
| EPI319998 | NA (N1) | Barbados | 2011-Jan-12 | A/Barbados/104/2011 | Caribbean Epidemiology Center | Centers for Disease Control and Prevention |
| EPI497719 | NA (N1) | Barbados | 2013-Sep-19 | A/Barbados/3024/2013 | Caribbean Epidemiology Center | Centers for Disease Control and Prevention |
| EPI216729 | NA (N1) | Belize | 2009-Jul-28 | A/Belize/7121/2009 | Caribbean Epidemiology Center | Centers for Disease Control and Prevention |
| EPI231290 | NA (N1) | Belize | 2009-Oct-08 | A/Belize/8756/2009 | Caribbean Epidemiology Center | Centers for Disease Control and Prevention |
| EPI295491 | NA (N1) | Belize | 2010-Aug-11 | A/Belize/3342/2010 | Caribbean Epidemiology Center | Centers for Disease Control and Prevention |
| EPI394846 | NA (N1) | Belize | 2012-Jan-01 | A/Belize/334/2012 | Caribbean Epidemiology Center | Centers for Disease Control and Prevention |
| EPI497710 | NA (N1) | Belize | 2013-Sep-23 | A/Belize/3161/2013 | Caribbean Epidemiology Center | Centers for Disease Control and Prevention |
| EPI186267 | NA (N1) | Bermuda | 2009-Jun-03 | A/Bermuda/4287/2009 | Caribbean Epidemiology Center | Centers for Disease Control and Prevention |
| EPI227627 | NA (N1) | Bermuda | 2009-Oct-23 | A/Bermuda/9261/2009 | Caribbean Epidemiology Center | Centers for Disease Control and Prevention |
| EPI320004 | NA (N1) | Cayman Islands | 2011-Jan-14 | A/Cayman Islands/284/2011 | Caribbean Epidemiology Center | Centers for Disease Control and Prevention |
| EPI181742 | NA (N1) | Costa Rica | 2009-Apr-28 | A/Costa Rica/4314/2009 | Laboratorio Nacional de Influenza | Centers for Disease Control and Prevention |
| EPI227649 | NA (N1) | Costa Rica | 2009-Jul-13 | A/Costa Rica/3149/2009 | Laboratorio Nacional de Influenza | Centers for Disease Control and Prevention |
| EPI216774 | NA (N1) | Costa Rica | 2009-Jul-22 | A/Costa Rica/4635/2009 | Laboratorio Nacional de Influenza | Centers for Disease Control and Prevention |
| EPI295455 | NA (N1) | Costa Rica | 2010-Jul-16 | A/Costa Rica/6692/2010 | Laboratorio Nacional de Influenza | Centers for Disease Control and Prevention |
| EPI307812 | NA (N1) | Costa Rica | 2010-Nov-01 | A/swine/Costa Rica/000125-3/2010 |  | Other Database Import |
| EPI307815 | NA (N1) | Costa Rica | 2010-Nov-01 | A/swine/Costa Rica/000125-14/2010 |  | Other Database Import |
| EPI307818 | NA (N1) | Costa Rica | 2010-Nov-01 | A/swine/Costa Rica/000125-15/2010 |  | Other Database Import |
| EPI307824 | NA (N1) | Costa Rica | 2010-Nov-01 | A/swine/Costa Rica/000125-19/2010 |  | Other Database Import |
| EPI350002 | NA (N1) | Costa Rica | 2011-Oct-11 | A/Costa Rica/8107/2011 | Laboratorio Nacional de Influenza | Centers for Disease Control and Prevention |
| EPI348170 | NA (N1) | Costa Rica | 2011-Oct-30 | A/Costa Rica/6796/2011 | Laboratorio Nacional de Influenza | Centers for Disease Control and Prevention |
| EPI376364 | NA (N1) | Costa Rica | 2012-Jan-17 | A/Costa Rica/4023/2012 | Laboratorio Nacional de Influenza | Centers for Disease Control and Prevention |
| EPI397471 | NA (N1) | Costa Rica | 2012-Jun-24 | A/Costa Rica/6288/2012 | Laboratorio Nacional de Influenza | Centers for Disease Control and Prevention |
| EPI485767 | NA (N1) | Costa Rica | 2013-Jun-10 | A/Costa Rica/7069/2013 | Laboratorio Nacional de Influenza | Centers for Disease Control and Prevention |
| EPI484963 | NA (N1) | Costa Rica | 2013-Jun-18 | A/Costa Rica/8288/2013 | Laboratorio Nacional de Influenza | Centers for Disease Control and Prevention |
| EPI484981 | NA (N1) | Costa Rica | 2013-May-30 | A/Costa Rica/5790/2013 | Laboratorio Nacional de Influenza | Centers for Disease Control and Prevention |
| EPI335975 | NA (N1) | Cuba | 2010-Nov-01 | A/swine/Villa Clara/84/2010 |  | Other Database Import |
| EPI338290 | NA (N1) | Cuba | 2010-Nov-01 | A/swine/Pinar del Rio/3/2010 |  | Other Database Import |
| EPI338308 | NA (N1) | Cuba | 2010-Nov-01 | A/swine/Holguin/121/2010 |  | Other Database Import |
| EPI247262 | NA (N1) | Dominican Republic | 2009-Dec-15 | A/Dominican Republic/3768/2009 | Laboratorio de Investigacion / Centro de Educacion Medica y Amistad Dominico Japones (CEMADOJA) | Centers for Disease Control and Prevention |
| EPI277659 | NA (N1) | Dominican Republic | 2009-Jun-10 | A/Santo Domingo/WR1072T/2009 |  | Other Database Import |
| EPI186659 | NA (N1) | Dominican Republic | 2009-May-26 | A/Santo Domingo/565T/2009 |  | Other Database Import |
| EPI231304 | NA (N1) | Dominican Republic | 2009-Oct-23 | A/Dominica/9250/2009 | Caribbean Epidemiology Center | Centers for Disease Control and Prevention |
| EPI335803 | NA (N1) | Dominican Republic | 2011-Jul-18 | A/Dominican Republic/5542/2011 | Laboratorio de Investigacion / Centro de Educacion Medica y Amistad Dominico Japones (CEMADOJA) | Centers for Disease Control and Prevention |
| EPI320010 | NA (N1) | Dominican Republic | 2011-Mar-28 | A/Dominican Republic/5059/2011 | Laboratorio de Investigacion / Centro de Educacion Medica y Amistad Dominico Japones (CEMADOJA) | Centers for Disease Control and Prevention |
| EPI376380 | NA (N1) | Dominican Republic | 2012-Feb-21 | A/Dominican Republic/6188/2012 | Laboratorio de Investigacion / Centro de Educacion Medica y Amistad Dominico Japones (CEMADOJA) | Centers for Disease Control and Prevention |
| EPI395237 | NA (N1) | Dominican Republic | 2012-Jun-08 | A/Dominican Republic/6551/2012 | Laboratorio de Investigacion / Centro de Educacion Medica y Amistad Dominico Japones (CEMADOJA) | Centers for Disease Control and Prevention |
| EPI396138 | NA (N1) | Dominican Republic | 2012-May-28 | A/Dominican Republic/6493/2012 | Laboratorio de Investigacion / Centro de Educacion Medica y Amistad Dominico Japones (CEMADOJA) | Centers for Disease Control and Prevention |
| EPI477421 | NA (N1) | Dominican Republic | 2013-Jun-05 | A/Dominican Republic/7548/2013 | Laboratorio de Investigacion / Centro de Educacion Medica y Amistad Dominico Japones (CEMADOJA) | Centers for Disease Control and Prevention |
| EPI467199 | NA (N1) | Dominican Republic | 2013-Jun-10 | A/Dominican Republic/7703/2013 | Laboratorio de Investigacion / Centro de Educacion Medica y Amistad Dominico Japones (CEMADOJA) | Centers for Disease Control and Prevention |
| EPI477444 | NA (N1) | Dominican Republic | 2013-Jun-15 | A/Dominican Republic/7626/2013 | Laboratorio de Investigacion / Centro de Educacion Medica y Amistad Dominico Japones (CEMADOJA) | Centers for Disease Control and Prevention |
| EPI457455 | NA (N1) | Dominican Republic | 2013-May-01 | A/Dominican Republic/7291/2013 | Laboratorio de Investigacion / Centro de Educacion Medica y Amistad Dominico Japones (CEMADOJA) | Centers for Disease Control and Prevention |
| EPI179076 | NA (N1) | El Salvador | 2009-Apr-26 | A/El Salvador/213/2009 | Contiguo a Hospital Rosales | Centers for Disease Control and Prevention |
| EPI216821 | NA (N1) | El Salvador | 2009-Jul-14 | A/El Salvador/1553/2009 | Contiguo a Hospital Rosales | Centers for Disease Control and Prevention |
| EPI216836 | NA (N1) | El Salvador | 2009-Jul-16 | A/El Salvador/1644/2009 | Contiguo a Hospital Rosales | Centers for Disease Control and Prevention |
| EPI215400 | NA (N1) | El Salvador | 2009-Jun-09 | A/San Salvador/WR0167N/2009 |  | Other Database Import |
| EPI183130 | NA (N1) | El Salvador | 2009-May-13 | A/El Salvador/351/2009 | Contiguo a Hospital Rosales | Centers for Disease Control and Prevention |
| EPI295398 | NA (N1) | El Salvador | 2010-Jul-13 | A/El Salvador/798/2010 | Contiguo a Hospital Rosales | Centers for Disease Control and Prevention |
| EPI278887 | NA (N1) | El Salvador | 2010-May-26 | A/El Salvador/455/2010 | Contiguo a Hospital Rosales | Centers for Disease Control and Prevention |
| EPI378077 | NA (N1) | El Salvador | 2012-Apr-17 | A/El Salvador/530/2012 | Contiguo a Hospital Rosales | Centers for Disease Control and Prevention |
| EPI484973 | NA (N1) | El Salvador | 2013-Jul-05 | A/El Salvador/1396/2013 | Contiguo a Hospital Rosales | Centers for Disease Control and Prevention |
| EPI534019 | NA (N1) | El Salvador | 2013-Nov-04 | A/El Salvador/2260/2013 | Contiguo a Hospital Rosales | Centers for Disease Control and Prevention |
| EPI504751 | NA (N1) | El Salvador | 2013-Nov-28 | A/El Salvador/2388/2013 | Contiguo a Hospital Rosales | Centers for Disease Control and Prevention |
| EPI231311 | NA (N1) | Grenada | 2009-Oct-23 | A/Grenada/9302/2009 | Caribbean Epidemiology Center | Centers for Disease Control and Prevention |
| EPI194156 | NA (N1) | Guatemala | 2009-Jun-02 | A/Guatemala/1039/2009 | Laboratorio Nacional De Salud Guatemala | Centers for Disease Control and Prevention |
| EPI347040 | NA (N1) | Guatemala | 2009-Oct-26 | A/Guatemala/4752/2009 | Laboratorio Nacional De Salud Guatemala | Centers for Disease Control and Prevention |
| EPI231314 | NA (N1) | Guatemala | 2009-Sep-15 | A/Guatemala/4360/2009 | Laboratorio Nacional De Salud Guatemala | Centers for Disease Control and Prevention |
| EPI278862 | NA (N1) | Guatemala | 2010-Apr-28 | A/Guatemala/358/2010 | Laboratorio Nacional De Salud Guatemala | Centers for Disease Control and Prevention |
| EPI295437 | NA (N1) | Guatemala | 2010-Aug-27 | A/Guatemala/287/2010 | Laboratorio Nacional De Salud Guatemala | Centers for Disease Control and Prevention |
| EPI273936 | NA (N1) | Guatemala | 2010-Jan-01 | A/Guatemala/127/2010 |  | Centers for Disease Control and Prevention |
| EPI362990 | NA (N1) | Guatemala | 2012-Feb-12 | A/Guatemala/18/2012 | Laboratorio Nacional De Salud Guatemala | Centers for Disease Control and Prevention |
| EPI216856 | NA (N1) | Haiti | 2009-Aug-12 | A/Haiti/265/2009 | Laboratoire National de Sante Publique | Centers for Disease Control and Prevention |
| EPI278011 | NA (N1) | Haiti | 2009-Nov-13 | A/Port Au Prince/WR2294T/2009 |  | Other Database Import |
| EPI239673 | NA (N1) | Haiti | 2009-Oct-19 | A/Haiti/534/2009 | Laboratoire National de Sante Publique | Centers for Disease Control and Prevention |
| EPI465087 | NA (N1) | Haiti | 2013-Jun-02 | A/Haiti/1790/2013 | Caribbean Epidemiology Center | Centers for Disease Control and Prevention |
| EPI465474 | NA (N1) | Haiti | 2013-Jun-02 | A/Haiti/1792/2013 | Caribbean Epidemiology Center | Centers for Disease Control and Prevention |
| EPI465402 | NA (N1) | Haiti | 2013-Jun-18 | A/Haiti/2030/2013 | Caribbean Epidemiology Center | Centers for Disease Control and Prevention |
| EPI465471 | NA (N1) | Haiti | 2013-Jun-20 | A/Haiti/2036/2013 | Caribbean Epidemiology Center | Centers for Disease Control and Prevention |
| EPI191949 | NA (N1) | Honduras | 2009-Jun-17 | A/Honduras/2039/2009 | Laboratorio Nacional de Virologia | Centers for Disease Control and Prevention |
| EPI216862 | NA (N1) | Honduras | 2009-Jun-25 | A/Honduras/2188/2009 | Laboratorio Nacional de Virologia | Centers for Disease Control and Prevention |
| EPI273770 | NA (N1) | Honduras | 2010-May-19 | A/Honduras/6132/2010 | Laboratorio Nacional de Virologia | Centers for Disease Control and Prevention |
| EPI394851 | NA (N1) | Honduras | 2012-Jun-14 | A/Honduras/9720/2012 | Laboratorio Nacional de Virologia | Centers for Disease Control and Prevention |
| EPI394739 | NA (N1) | Honduras | 2012-Jun-26 | A/Honduras/9746/2012 | Laboratorio Nacional de Virologia | Centers for Disease Control and Prevention |
| EPI273919 | NA (N1) | Jamaica | 2009-Jul-07 | A/Jamaica/6261/2009 | University of the West Indies | Centers for Disease Control and Prevention |
| EPI186265 | NA (N1) | Jamaica | 2009-Jun-06 | A/Jamaica/4423/2009 | Caribbean Epidemiology Center | Centers for Disease Control and Prevention |
| EPI391295 | NA (N1) | Jamaica | 2012-Feb-13 | A/Jamaica/764/2012 | University of the West Indies | Centers for Disease Control and Prevention |
| EPI492854 | NA (N1) | Jamaica | 2013-Oct-30 | A/Jamaica/198/2013 | University of the West Indies | Centers for Disease Control and Prevention |
| EPI230499 | NA (N1) | Mexico | 2009-Apr-01 | A/swine/4/Mexico/2009 |  | Other Database Import |
| EPI176588 | NA (N1) | Mexico | 2009-Apr-14 | A/Mexico/4482/2009 | Laboratorio de Virus Respiratorio | Centers for Disease Control and Prevention |
| EPI179101 | NA (N1) | Mexico | 2009-Apr-20 | A/Mexico/4575/2009 | Laboratorio de Virus Respiratorio | Centers for Disease Control and Prevention |
| EPI186277 | NA (N1) | Mexico | 2009-Apr-20 | A/Mexico/4635/2009 | Laboratorio de Virus Respiratorio | Centers for Disease Control and Prevention |
| EPI180757 | NA (N1) | Mexico | 2009-Apr-25 | A/Mexico/47N/2009 |  | Other Database Import |
| EPI190865 | NA (N1) | Mexico | 2009-Apr-25 | A/Mexico/48N/2009 |  | Other Database Import |
| EPI256697 | NA (N1) | Mexico | 2009-Jul-06 | A/Mexico city/CIA2/2009 |  | Other Database Import |
| EPI256704 | NA (N1) | Mexico | 2009-Jul-06 | A/Mexico city/CIA3/2009 |  | Other Database Import |
| EPI231328 | NA (N1) | Mexico | 2009-Jul-10 | A/Mexico/2466/2009 | Laboratorio de Virus Respiratorio | Centers for Disease Control and Prevention |
| EPI267002 | NA (N1) | Mexico | 2009-May-02 | A/Mexico City/026/2009 |  | Other Database Import |
| EPI217524 | NA (N1) | Mexico | 2009-May-08 | A/Mexico City/005/2009 |  | Other Database Import |
| EPI217596 | NA (N1) | Mexico | 2009-May-09 | A/Mexico City/015/2009 |  | Other Database Import |
| EPI217604 | NA (N1) | Mexico | 2009-May-09 | A/Mexico City/017/2009 |  | Other Database Import |
| EPI217612 | NA (N1) | Mexico | 2009-May-10 | A/Mexico City/018/2009 |  | Other Database Import |
| EPI256740 | NA (N1) | Mexico | 2009-May-16 | A/Mexico city/CIA9/2009 |  | Other Database Import |
| EPI277979 | NA (N1) | Mexico | 2009-Nov-09 | A/Mexico City/WR1747N/2009 |  | Other Database Import |
| EPI277995 | NA (N1) | Mexico | 2009-Nov-19 | A/Mexico City/WR1765N/2009 |  | Other Database Import |
| EPI273924 | NA (N1) | Mexico | 2009-Oct-18 | A/Mexico/4178/2009 | Laboratorio de Virus Respiratorio | Centers for Disease Control and Prevention |
| EPI243932 | NA (N1) | Mexico | 2009-Oct-26 | A/Mexico/1138/2009 | Laboratorio de Virus Respiratorio | Centers for Disease Control and Prevention |
| EPI215328 | NA (N1) | Mexico | 2009-Sep-09 | A/Mexico City/WR1310N/2009 |  | Other Database Import |
| EPI277963 | NA (N1) | Mexico | 2009-Sep-18 | A/Mexico City/WR1706T/2009 |  | Other Database Import |
| EPI278877 | NA (N1) | Mexico | 2010-Apr-07 | A/Mexico/2880/2010 | Laboratorio de Virus Respiratorio | Centers for Disease Control and Prevention |
| EPI273878 | NA (N1) | Mexico | 2010-Mar-01 | A/Mexico/1762/2010 | Laboratorio de Virus Respiratorio | Centers for Disease Control and Prevention |
| EPI353436 | NA (N1) | Mexico | 2011-Dec-12 | A/Mexico/3723/2011 | Laboratorio de Virus Respiratorio | Centers for Disease Control and Prevention |
| EPI355515 | NA (N1) | Mexico | 2011-Dec-12 | A/Mexico/3752/2011 | Laboratorio de Virus Respiratorio | Centers for Disease Control and Prevention |
| EPI353404 | NA (N1) | Mexico | 2011-Dec-14 | A/Mexico/3720/2011 | Laboratorio de Virus Respiratorio | Centers for Disease Control and Prevention |
| EPI353410 | NA (N1) | Mexico | 2011-Dec-30 | A/Mexico/56/2011 | Laboratorio de Virus Respiratorio | Centers for Disease Control and Prevention |
| EPI320044 | NA (N1) | Mexico | 2011-Feb-22 | A/Mexico/1658/2011 | Laboratorio de Virus Respiratorio | Centers for Disease Control and Prevention |
| EPI331209 | NA (N1) | Mexico | 2011-Mar-15 | A/Mexico/2208/2011 | Laboratorio de Virus Respiratorio | Centers for Disease Control and Prevention |
| EPI320047 | NA (N1) | Mexico | 2011-Mar-22 | A/Mexico/1946/2011 | Laboratorio de Virus Respiratorio | Centers for Disease Control and Prevention |
| EPI353442 | NA (N1) | Mexico | 2012-Jan-02 | A/Mexico/254/2012 | Laboratorio de Virus Respiratorio | Centers for Disease Control and Prevention |
| EPI353401 | NA (N1) | Mexico | 2012-Jan-08 | A/Mexico/210/2012 | Laboratorio de Virus Respiratorio | Centers for Disease Control and Prevention |
| EPI366298 | NA (N1) | Mexico | 2012-Jan-19 | A/Mexico/689/2012 | Laboratorio de Virus Respiratorio | Centers for Disease Control and Prevention |
| EPI366289 | NA (N1) | Mexico | 2012-Jan-21 | A/Mexico/1818/2012 | Laboratorio de Virus Respiratorio | Centers for Disease Control and Prevention |
| EPI366286 | NA (N1) | Mexico | 2012-Jan-26 | A/Mexico/1474/2012 | Laboratorio de Virus Respiratorio | Centers for Disease Control and Prevention |
| EPI508442 | NA (N1) | Mexico | 2013-Aug-31 | A/Mexico/2733/2013 | Laboratorio de Virus Respiratorio | Centers for Disease Control and Prevention |
| EPI504790 | NA (N1) | Mexico | 2013-Dec-27 | A/Mexico/06/2013 | Laboratorio de Virus Respiratorio | Centers for Disease Control and Prevention |
| EPI467232 | NA (N1) | Mexico | 2013-Jul-13 | A/Mexico/2410/2013 | Laboratorio de Virus Respiratorio | Centers for Disease Control and Prevention |
| EPI508466 | NA (N1) | Mexico | 2013-Oct-08 | A/Mexico/3093/2013 | Laboratorio de Virus Respiratorio | Centers for Disease Control and Prevention |
| EPI503826 | NA (N1) | Mexico | 2013-Oct-21 | A/Mexico/3280/2013 | Laboratorio de Virus Respiratorio | Centers for Disease Control and Prevention |
| EPI177291 | NA (N1) | Netherlands | 2009-Jan-01 | A/Netherlands/602/2009 |  | Erasmus University of Rotterdam |
| EPI249118 | NA (N1) | Nicaragua | 2009-Aug-15 | A/Managua/473.02/2009 |  | Other Database Import |
| EPI249584 | NA (N1) | Nicaragua | 2009-Aug-18 | A/Managua/2323.02/2009 |  | Other Database Import |
| EPI249592 | NA (N1) | Nicaragua | 2009-Aug-18 | A/Managua/3435.01/2009 |  | Other Database Import |
| EPI227689 | NA (N1) | Nicaragua | 2009-Aug-25 | A/Managua/6502/2009 | Laboratorio de Virologia, Direccion de Microbiologia | Centers for Disease Control and Prevention |
| EPI275083 | NA (N1) | Nicaragua | 2009-Jul-21 | A/Managua/5364.01/2009 |  | Other Database Import |
| EPI249640 | NA (N1) | Nicaragua | 2009-Jun-15 | A/Managua/4935.03/2009 |  | Other Database Import |
| EPI190857 | NA (N1) | Nicaragua | 2009-Jun-26 | A/Managua/0536N/2009 |  | Other Database Import |
| EPI249616 | NA (N1) | Nicaragua | 2009-Jun-26 | A/Managua/462.01/2009 |  | Other Database Import |
| EPI273843 | NA (N1) | Nicaragua | 2010-Apr-01 | A/Nicaragua/322/2010 | Laboratorio de Virologia, Direccion de Microbiologia | Centers for Disease Control and Prevention |
| EPI346506 | NA (N1) | Nicaragua | 2011-Oct-10 | A/Managua/748/2011 | Laboratorio de Virologia, Direccion de Microbiologia | Centers for Disease Control and Prevention |
| EPI465278 | NA (N1) | Nicaragua | 2013-Apr-20 | A/Managua/687/2013 | Laboratorio de Virologia, Direccion de Microbiologia | Centers for Disease Control and Prevention |
| EPI465461 | NA (N1) | Nicaragua | 2013-Jun-14 | A/Managua/30074.01/2013 | Laboratorio de Virologia, Direccion de Microbiologia | Centers for Disease Control and Prevention |
| EPI465463 | NA (N1) | Nicaragua | 2013-Jun-19 | A/Matagalpa/1076/2013 | Laboratorio de Virologia, Direccion de Microbiologia | Centers for Disease Control and Prevention |
| EPI183240 | NA (N1) | Panama | 2009-May-11 | A/Panama/302869/2009 | Instituto Conmemorativo Gorgas de Estudios de la Salud | Centers for Disease Control and Prevention |
| EPI278851 | NA (N1) | Panama | 2010-Jun-07 | A/Panama/307168/2010 | Instituto Conmemorativo Gorgas de Estudios de la Salud | Centers for Disease Control and Prevention |
| EPI335821 | NA (N1) | Panama | 2011-Jun-10 | A/Panama/309335/2011 | Instituto Conmemorativo Gorgas de Estudios de la Salud | Centers for Disease Control and Prevention |
| EPI394748 | NA (N1) | Panama | 2012-Jun-05 | A/Panama/310521/2012 | Instituto Conmemorativo Gorgas de Estudios de la Salud | Centers for Disease Control and Prevention |
| EPI394751 | NA (N1) | Panama | 2012-Jun-26 | A/Panama/310630/2012 | Instituto Conmemorativo Gorgas de Estudios de la Salud | Centers for Disease Control and Prevention |
| EPI467410 | NA (N1) | Panama | 2013-Jun-13 | A/Panama/313106/2013 | Instituto Conmemorativo Gorgas de Estudios de la Salud | Centers for Disease Control and Prevention |
| EPI467380 | NA (N1) | Panama | 2013-Jun-25 | A/Panama/313202/2013 | Instituto Conmemorativo Gorgas de Estudios de la Salud | Centers for Disease Control and Prevention |
| EPI240402 | NA (N1) | Puerto Rico | 2009-Dec-06 | A/Puerto Rico/51/2009 | Puerto Rico Department of Health | Centers for Disease Control and Prevention |
| EPI254050 | NA (N1) | Puerto Rico | 2009-Dec-18 | A/Puerto Rico/20/2009 | Puerto Rico Department of Health | Centers for Disease Control and Prevention |
| EPI346509 | NA (N1) | Puerto Rico | 2011-Aug-03 | A/Puerto Rico/04/2011 | Puerto Rico Department of Health | Centers for Disease Control and Prevention |
| EPI366313 | NA (N1) | Puerto Rico | 2011-Aug-18 | A/Puerto Rico/25/2011 | Puerto Rico Department of Health | Centers for Disease Control and Prevention |
| EPI368448 | NA (N1) | Puerto Rico | 2011-Dec-29 | A/Puerto Rico/33/2011 | Puerto Rico Department of Health | Centers for Disease Control and Prevention |
| EPI366310 | NA (N1) | Puerto Rico | 2011-Jul-19 | A/Puerto Rico/09/2011 | Puerto Rico Department of Health | Centers for Disease Control and Prevention |
| EPI366307 | NA (N1) | Puerto Rico | 2011-May-31 | A/Puerto Rico/05/2011 | Puerto Rico Department of Health | Centers for Disease Control and Prevention |
| EPI349361 | NA (N1) | Puerto Rico | 2011-Nov-21 | A/Puerto Rico/8233/2011 | Puerto Rico Department of Health | Centers for Disease Control and Prevention |
| EPI347555 | NA (N1) | Puerto Rico | 2011-Oct-22 | A/Puerto Rico/21/2011 | Puerto Rico Department of Health | Centers for Disease Control and Prevention |
| EPI368446 | NA (N1) | Puerto Rico | 2012-Feb-14 | A/Puerto Rico/01/2012 | Puerto Rico Department of Health | Centers for Disease Control and Prevention |
| EPI398006 | NA (N1) | Puerto Rico | 2012-Jul-03 | A/Puerto Rico/43/2012 | Puerto Rico Department of Health | Centers for Disease Control and Prevention |
| EPI391292 | NA (N1) | Puerto Rico | 2012-Jun-07 | A/Puerto Rico/06/2012 | Puerto Rico Department of Health | Centers for Disease Control and Prevention |
| EPI392906 | NA (N1) | Puerto Rico | 2012-Jun-12 | A/Puerto Rico/20/2012 | Puerto Rico Department of Health | Centers for Disease Control and Prevention |
| EPI508440 | NA (N1) | Puerto Rico | 2013-Dec-16 | A/Puerto Rico/21/2013 | Puerto Rico Department of Health | Centers for Disease Control and Prevention |
| EPI465069 | NA (N1) | Puerto Rico | 2013-Jun-14 | A/Puerto Rico/01/2013 | Puerto Rico Department of Health | Centers for Disease Control and Prevention |
| EPI486391 | NA (N1) | Puerto Rico | 2013-Oct-01 | A/Puerto Rico/18/2013 | Puerto Rico Department of Health | Centers for Disease Control and Prevention |
| EPI217090 | NA (N1) | Saint Lucia | 2009-Jul-31 | A/Saint Lucia/7178/2009 | Caribbean Epidemiology Center | Centers for Disease Control and Prevention |
| EPI231340 | NA (N1) | Saint Lucia | 2009-Sep-25 | A/St. Lucia/9333/2009 | Caribbean Epidemiology Center | Centers for Disease Control and Prevention |
| EPI497716 | NA (N1) | Saint Vincent and the Grenadines | 2013-Oct-07 | A/St. Vincent and Grenadines/3291/2013 | Caribbean Epidemiology Center | Centers for Disease Control and Prevention |
| EPI497713 | NA (N1) | Saint Vincent and the Grenadines | 2013-Oct-08 | A/St. Vincent and Grenadines/3292/2013 | Caribbean Epidemiology Center | Centers for Disease Control and Prevention |
| EPI186301 | NA (N1) | Trinidad and Tobago | 2009-Jan-01 | A/Trinidad/4601/2009 |  | Centers for Disease Control and Prevention |
| EPI231349 | NA (N1) | Trinidad and Tobago | 2009-Oct-22 | A/Trinidad/9184/2009 | Caribbean Epidemiology Center | Centers for Disease Control and Prevention |
| EPI335836 | NA (N1) | Trinidad and Tobago | 2011-Jul-08 | A/Trinidad/1648/2011 | Caribbean Epidemiology Center | Centers for Disease Control and Prevention |
| EPI460141 | NA (N1) | Trinidad and Tobago | 2013-Mar-01 | A/Trinidad/979/2013 | Caribbean Epidemiology Center | Centers for Disease Control and Prevention |
| EPI459773 | NA (N1) | Trinidad and Tobago | 2013-Mar-13 | A/Trinidad/982/2013 | Caribbean Epidemiology Center | Centers for Disease Control and Prevention |
| EPI497648 | NA (N1) | Trinidad and Tobago | 2013-Oct-24 | A/Trinidad/3568/2013 | Caribbean Epidemiology Center | Centers for Disease Control and Prevention |
| EPI231352 | NA (N1) | Turks and Caicos Islands | 2009-Oct-13 | A/Turks And Caicos/9060/2009 | Caribbean Epidemiology Center | Centers for Disease Control and Prevention |
| EPI320154 | NA (N1) | Turks and Caicos Islands | 2011-Jan-25 | A/Turks and Caicos/211/2011 | Caribbean Epidemiology Center | Centers for Disease Control and Prevention |
| EPI221062 | NA (N1) | United States | 2009-Apr-09 | A/California/07/2009 | Naval Health Research Center | Centers for Disease Control and Prevention |
| EPI341572 | NA (N1) | United States | 2009-Jan-01 | A/California/04/2009 |  | Other Database Import |
| EPI193943 | NA (N2) | Anguilla | 2009-Jun-10 | A/Anguilla/4711/2009 | Caribbean Epidemiology Center | Centers for Disease Control and Prevention |
| EPI319647 | NA (N2) | Anguilla | 2011-Jan-24 | A/Anguilla/273/2011 | Caribbean Epidemiology Center | Centers for Disease Control and Prevention |
| EPI459801 | NA (N2) | Anguilla | 2013-Jan-10 | A/Anguilla/104/2013 | Caribbean Epidemiology Center | Centers for Disease Control and Prevention |
| EPI176961 | NA (N2) | Australia | 2007-Jan-01 | A/Brisbane/10/2007 |  | Other Database Import |
| EPI211335 | NA (N2) | Australia | 2009-Jan-01 | A/Perth/16/2009 | WHO Collaborating Centre for Reference and Research on Influenza | Centers for Disease Control and Prevention |
| EPI513285 | NA (N2) | Australia | 2011-Oct-24 | A/Victoria/361/2011 | WHO Collaborating Centre for Reference and Research on Influenza | Centers for Disease Control and Prevention |
| EPI279978 | NA (N2) | Barbados | 2010-Aug-11 | A/Barbados/170/2010 | Public Health Laboratory | Centers for Disease Control and Prevention |
| EPI459834 | NA (N2) | Barbados | 2013-Feb-13 | A/Barbados/4326/2013 | Caribbean Epidemiology Center | Centers for Disease Control and Prevention |
| EPI459831 | NA (N2) | Barbados | 2013-Jan-12 | A/Barbados/1031/2013 | Caribbean Epidemiology Center | Centers for Disease Control and Prevention |
| EPI295198 | NA (N2) | Belize | 2010-Jan-01 | A/Belize/3715/2010 |  | Centers for Disease Control and Prevention |
| EPI394899 | NA (N2) | Bermuda | 2012-Jun-13 | A/Bermuda/1915/2012 | Caribbean Epidemiology Center | Centers for Disease Control and Prevention |
| EPI459843 | NA (N2) | Bermuda | 2013-Jan-28 | A/Bermuda/521/2013 | Caribbean Epidemiology Center | Centers for Disease Control and Prevention |
| EPI155502 | NA (N2) | Costa Rica | 2007-Jan-05 | A/Costa Rica/176/2007 |  | Centers for Disease Control and Prevention |
| EPI172433 | NA (N2) | Costa Rica | 2008-Jun-19 | A/Costa Rica/7172/2008 |  | Centers for Disease Control and Prevention |
| EPI232480 | NA (N2) | Costa Rica | 2009-Jul-24 | A/Costa Rica/5023/2009 | Laboratorio Nacional de Influenza | Centers for Disease Control and Prevention |
| EPI211318 | NA (N2) | Costa Rica | 2009-Jun-05 | A/Costa Rica/5179/2009 | Laboratorio Nacional de Influenza | Centers for Disease Control and Prevention |
| EPI291555 | NA (N2) | Costa Rica | 2010-Jul-16 | A/Costa Rica/6696/2010 | Laboratorio Nacional de Influenza | Centers for Disease Control and Prevention |
| EPI349760 | NA (N2) | Costa Rica | 2011-Nov-11 | A/Costa Rica/8211/2011 | Laboratorio Nacional de Influenza | Centers for Disease Control and Prevention |
| EPI397143 | NA (N2) | Costa Rica | 2012-Jul-17 | A/Costa Rica/9093/2012 | Laboratorio Nacional de Influenza | Centers for Disease Control and Prevention |
| EPI484548 | NA (N2) | Costa Rica | 2013-Jul-12 | A/Costa Rica/2932/2013 | Laboratorio Nacional de Influenza | Centers for Disease Control and Prevention |
| EPI484732 | NA (N2) | Costa Rica | 2013-Jul-23 | A/Costa Rica/4009/2013 | Laboratorio Nacional de Influenza | Centers for Disease Control and Prevention |
| EPI485799 | NA (N2) | Costa Rica | 2013-Jul-26 | A/Costa Rica/4700/2013 | Laboratorio Nacional de Influenza | Centers for Disease Control and Prevention |
| EPI155855 | NA (N2) | Dominica | 2007-Feb-25 | A/Dominican Republic/2896/2007 |  | Centers for Disease Control and Prevention |
| EPI155857 | NA (N2) | Dominica | 2007-Feb-25 | A/Dominica/2898/2007 |  | Centers for Disease Control and Prevention |
| EPI391244 | NA (N2) | Dominica | 2012-Mar-08 | A/Dominica/616/2012 | Caribbean Epidemiology Center | Centers for Disease Control and Prevention |
| EPI459804 | NA (N2) | Dominica | 2013-Jan-21 | A/Dominica/653/2013 | Caribbean Epidemiology Center | Centers for Disease Control and Prevention |
| EPI498066 | NA (N2) | Dominica | 2013-Oct-07 | A/Dominica/3307/2013 | Caribbean Epidemiology Center | Centers for Disease Control and Prevention |
| EPI211267 | NA (N2) | Dominican Republic | 2009-May-25 | A/Dominican Republic/988/2009 | Laboratorio de Investigacion / Centro de Educacion Medica y Amistad Dominico Japones (CEMADOJA) | Centers for Disease Control and Prevention |
| EPI254585 | NA (N2) | Dominican Republic | 2009-Nov-12 | A/Dominican Republic/3668/2009 | Laboratorio de Investigacion / Centro de Educacion Medica y Amistad Dominico Japones (CEMADOJA) | Centers for Disease Control and Prevention |
| EPI278765 | NA (N2) | Dominican Republic | 2010-Jul-20 | A/Dominican Republic/4319/2010 | Laboratorio de Investigacion / Centro de Educacion Medica y Amistad Dominico Japones (CEMADOJA) | Centers for Disease Control and Prevention |
| EPI376514 | NA (N2) | Dominican Republic | 2012-Apr-13 | A/Dominican Republic/6334/2012 | Laboratorio de Investigacion / Centro de Educacion Medica y Amistad Dominico Japones (CEMADOJA) | Centers for Disease Control and Prevention |
| EPI156056 | NA (N2) | El Salvador | 2007-Aug-09 | A/El Salvador/428/2007 |  | Centers for Disease Control and Prevention |
| EPI156058 | NA (N2) | El Salvador | 2007-Aug-15 | A/El Salvador/507/2007 |  | Centers for Disease Control and Prevention |
| EPI157580 | NA (N2) | El Salvador | 2007-Sep-03 | A/El Salvador/579/2007 |  | Centers for Disease Control and Prevention |
| EPI161998 | NA (N2) | El Salvador | 2007-Sep-03 | A/El Salvador/579/2007 |  | Centers for Disease Control and Prevention |
| EPI157586 | NA (N2) | El Salvador | 2007-Sep-06 | A/El Salvador/590/2007 |  | Centers for Disease Control and Prevention |
| EPI162004 | NA (N2) | El Salvador | 2007-Sep-06 | A/El Salvador/590/2007 |  | Centers for Disease Control and Prevention |
| EPI325875 | NA (N2) | El Salvador | 2010-Aug-11 | A/San Salvador/WRAIR3537T/2010 |  | Other Database Import |
| EPI295135 | NA (N2) | El Salvador | 2010-Aug-24 | A/El Salvador/1318/2010 | Contiguo a Hospital Rosales | Centers for Disease Control and Prevention |
| EPI278809 | NA (N2) | El Salvador | 2010-Jun-28 | A/El Salvador/635/2010 | Contiguo a Hospital Rosales | Centers for Disease Control and Prevention |
| EPI347485 | NA (N2) | El Salvador | 2011-Sep-21 | A/El Salvador/1513/2011 | Contiguo a Hospital Rosales | Centers for Disease Control and Prevention |
| EPI477436 | NA (N2) | El Salvador | 2013-Jul-04 | A/El Salvador/1307/2013 | Contiguo a Hospital Rosales | Centers for Disease Control and Prevention |
| EPI459840 | NA (N2) | El Salvador | 2013-Mar-18 | A/El Salvador/433/2013 | Contiguo a Hospital Rosales | Centers for Disease Control and Prevention |
| EPI342268 | NA (N2) | Guadeloupe | 2010-Nov-30 | A/Guadeloupe/200/2010 | National Influenza Center French Guiana and French Indies | Centers for Disease Control and Prevention |
| EPI342274 | NA (N2) | Guadeloupe | 2010-Nov-30 | A/Guadeloupe/201/2010 | National Influenza Center French Guiana and French Indies | Centers for Disease Control and Prevention |
| EPI309437 | NA (N2) | Guadeloupe | 2010-Oct-20 | A/Guadeloupe/129/2010 | National Influenza Center French Guiana and French Indies | Centers for Disease Control and Prevention |
| EPI157632 | NA (N2) | Guatemala | 2007-Aug-18 | A/Guatemala/7562/2007 |  | Centers for Disease Control and Prevention |
| EPI162057 | NA (N2) | Guatemala | 2007-Aug-18 | A/Guatemala/7562/2007 |  | Centers for Disease Control and Prevention |
| EPI163131 | NA (N2) | Guatemala | 2008-May-19 | A/Guatemala/494/2008 |  | Centers for Disease Control and Prevention |
| EPI163133 | NA (N2) | Guatemala | 2008-May-28 | A/Guatemala/545/2008 |  | Centers for Disease Control and Prevention |
| EPI193981 | NA (N2) | Guatemala | 2009-Jul-09 | A/Guatemala/29/2009 | Laboratorio Nacional De Salud Guatemala | Centers for Disease Control and Prevention |
| EPI193925 | NA (N2) | Guatemala | 2009-Jun-03 | A/Guatemala/1066/2009 | Laboratorio Nacional De Salud Guatemala | Centers for Disease Control and Prevention |
| EPI232440 | NA (N2) | Guatemala | 2009-Jun-14 | A/Guatemala/1839/2009 | Laboratorio Nacional De Salud Guatemala | Centers for Disease Control and Prevention |
| EPI211338 | NA (N2) | Guatemala | 2009-Jun-15 | A/Guatemala/1913/2009 | Laboratorio Nacional De Salud Guatemala | Centers for Disease Control and Prevention |
| EPI278774 | NA (N2) | Guatemala | 2010-Jul-13 | A/Guatemala/591/2010 | Laboratorio Nacional De Salud Guatemala | Centers for Disease Control and Prevention |
| EPI301070 | NA (N2) | Guatemala | 2010-Oct-05 | A/Guatemala/754/2010 | Laboratorio Nacional De Salud Guatemala | Centers for Disease Control and Prevention |
| EPI295094 | NA (N2) | Guatemala | 2010-Sep-02 | A/Guatemala/690/2010 | Laboratorio Nacional De Salud Guatemala | Centers for Disease Control and Prevention |
| EPI326290 | NA (N2) | Guatemala | 2011-Mar-02 | A/Guatemala/51/2011 | Laboratorio Nacional De Salud Guatemala | Centers for Disease Control and Prevention |
| EPI468140 | NA (N2) | Guatemala | 2013-Jul-09 | A/Guatemala/287/2013 | Laboratorio Nacional De Salud Guatemala | Centers for Disease Control and Prevention |
| EPI232462 | NA (N2) | Haiti | 2009-Jul-01 | A/Haiti/66/2009 | Laboratoire National de Sante Publique | Centers for Disease Control and Prevention |
| EPI157659 | NA (N2) | Honduras | 2007-Aug-26 | A/Honduras/5560/2007 |  | Centers for Disease Control and Prevention |
| EPI162060 | NA (N2) | Honduras | 2007-Aug-26 | A/Honduras/5560/2007 |  | Centers for Disease Control and Prevention |
| EPI163228 | NA (N2) | Honduras | 2007-Nov-18 | A/Honduras/1038/2007 |  | Centers for Disease Control and Prevention |
| EPI157665 | NA (N2) | Honduras | 2007-Oct-05 | A/Honduras/6374/2007 |  | Centers for Disease Control and Prevention |
| EPI162065 | NA (N2) | Honduras | 2007-Oct-05 | A/Honduras/6374/2007 |  | Centers for Disease Control and Prevention |
| EPI157671 | NA (N2) | Honduras | 2007-Oct-11 | A/Honduras/6453/2007 |  | Centers for Disease Control and Prevention |
| EPI162071 | NA (N2) | Honduras | 2007-Oct-11 | A/Honduras/6453/2007 |  | Centers for Disease Control and Prevention |
| EPI157663 | NA (N2) | Honduras | 2007-Oct-27 | A/Honduras/602/2007 |  | Centers for Disease Control and Prevention |
| EPI162063 | NA (N2) | Honduras | 2007-Oct-27 | A/Honduras/602/2007 |  | Centers for Disease Control and Prevention |
| EPI211350 | NA (N2) | Honduras | 2009-Jul-13 | A/Honduras/2426/2009 | Laboratorio Nacional de Virologia | Centers for Disease Control and Prevention |
| EPI193975 | NA (N2) | Honduras | 2009-Jun-30 | A/Honduras/2243/2009 | Laboratorio Nacional de Virologia | Centers for Disease Control and Prevention |
| EPI185777 | NA (N2) | Honduras | 2009-May-05 | A/Honduras/56/2009 | Laboratorio Nacional de Virologia | Centers for Disease Control and Prevention |
| EPI185780 | NA (N2) | Honduras | 2009-May-06 | A/Honduras/639/2009 | Laboratorio Nacional de Virologia | Centers for Disease Control and Prevention |
| EPI193898 | NA (N2) | Honduras | 2009-May-14 | A/Honduras/105/2009 | Laboratorio Nacional de Virologia | Centers for Disease Control and Prevention |
| EPI274452 | NA (N2) | Honduras | 2010-Apr-13 | A/Honduras/6065/2010 | Laboratorio Nacional de Virologia | Centers for Disease Control and Prevention |
| EPI295258 | NA (N2) | Honduras | 2010-Aug-17 | A/Honduras/6705/2010 | Laboratorio Nacional de Virologia | Centers for Disease Control and Prevention |
| EPI185783 | NA (N2) | Jamaica | 2009-Apr-28 | A/Jamaica/2970/2009 | Caribbean Epidemiology Center | Centers for Disease Control and Prevention |
| EPI492693 | NA (N2) | Jamaica | 2013-Oct-21 | A/Jamaica/200/2013 | University of the West Indies | Centers for Disease Control and Prevention |
| EPI157957 | NA (N2) | Martinique | 2007-Feb-09 | A/Martinique/12/2007 |  | Centers for Disease Control and Prevention |
| EPI162122 | NA (N2) | Martinique | 2007-Feb-09 | A/Martinique/12/2007 |  | Centers for Disease Control and Prevention |
| EPI378089 | NA (N2) | Martinique | 2012-May-07 | A/Martinique/201/2012 | National Influenza Center French Guiana and French Indies | Centers for Disease Control and Prevention |
| EPI155990 | NA (N2) | Mexico | 2007-May-03 | A/Mexico/2222/2007 |  | Centers for Disease Control and Prevention |
| EPI463956 | NA (N2) | Mexico | 2008-Dec-01 | A/Mexico/24014/2008 |  | Other Database Import |
| EPI463940 | NA (N2) | Mexico | 2008-Dec-02 | A/Mexico/24012/2008 |  | Other Database Import |
| EPI162129 | NA (N2) | Mexico | 2008-Jan-17 | A/Mexico/499/2008 |  | Centers for Disease Control and Prevention |
| EPI336098 | NA (N2) | Mexico | 2008-Jan-21 | A/Mexico/UASLP-013/2008 |  | Other Database Import |
| EPI463840 | NA (N2) | Mexico | 2008-Nov-01 | A/Mexico/24003/2008 |  | Other Database Import |
| EPI463895 | NA (N2) | Mexico | 2009-Apr-01 | A/Mexico/24008/2009 |  | Other Database Import |
| EPI464030 | NA (N2) | Mexico | 2009-Apr-27 | A/Mexico/24025/2009 |  | Other Database Import |
| EPI270306 | NA (N2) | Mexico | 2009-Aug-17 | A/Mexico/5824/2009 | Laboratorio de Virus Respiratorio | Centers for Disease Control and Prevention |
| EPI185798 | NA (N2) | Mexico | 2009-Mar-11 | A/Mexico/2779/2009 | Laboratorio de Virus Respiratorio | Centers for Disease Control and Prevention |
| EPI243568 | NA (N2) | Mexico | 2009-Sep-05 | A/Mexico/7880/2009 | Laboratorio de Virus Respiratorio | Centers for Disease Control and Prevention |
| EPI243562 | NA (N2) | Mexico | 2009-Sep-15 | A/Mexico/5270/2009 | Laboratorio de Virus Respiratorio | Centers for Disease Control and Prevention |
| EPI279987 | NA (N2) | Mexico | 2010-Aug-13 | A/Mexico/4056/2010 | Laboratorio de Virus Respiratorio | Centers for Disease Control and Prevention |
| EPI325947 | NA (N2) | Mexico | 2010-Dec-08 | A/Mexico City/WRAIR3578N/2010 |  | Other Database Import |
| EPI325963 | NA (N2) | Mexico | 2010-Dec-09 | A/Mexico City/WRAIR3579N/2010 |  | Other Database Import |
| EPI325971 | NA (N2) | Mexico | 2010-Dec-09 | A/Mexico City/WRAIR3579T/2010 |  | Other Database Import |
| EPI325915 | NA (N2) | Mexico | 2010-Dec-22 | A/Mexico City/WRAIR3570T/2010 |  | Other Database Import |
| EPI325803 | NA (N2) | Mexico | 2010-Jan-26 | A/Mexico City/WRAIR1752T/2010 |  | Other Database Import |
| EPI334632 | NA (N2) | Mexico | 2010-Nov-04 | A/Mexico/6998/2010 | Laboratorio de Virus Respiratorio | Centers for Disease Control and Prevention |
| EPI325931 | NA (N2) | Mexico | 2010-Nov-22 | A/Mexico City/WRAIR3571N/2010 |  | Other Database Import |
| EPI309386 | NA (N2) | Mexico | 2010-Oct-11 | A/Mexico/6605/2010 | Laboratorio de Virus Respiratorio | Centers for Disease Control and Prevention |
| EPI331239 | NA (N2) | Mexico | 2011-Apr-08 | A/Mexico/2554/2011 | Laboratorio de Virus Respiratorio | Centers for Disease Control and Prevention |
| EPI319722 | NA (N2) | Mexico | 2011-Feb-18 | A/Mexico/1664/2011 | Laboratorio de Virus Respiratorio | Centers for Disease Control and Prevention |
| EPI173818 | NA (N2) | Nicaragua | 2007-Aug-03 | A/Managua/4902.01/2007 |  | Other Database Import |
| EPI178232 | NA (N2) | Nicaragua | 2007-Jun-05 | A/Managua/4348.01/2007 |  | Other Database Import |
| EPI154140 | NA (N2) | Nicaragua | 2007-Jun-11 | A/Managua/28/2007 |  | Other Database Import |
| EPI162165 | NA (N2) | Nicaragua | 2007-Jun-13 | A/Managua/68.01/2007 |  | Centers for Disease Control and Prevention |
| EPI154068 | NA (N2) | Nicaragua | 2007-Jun-21 | A/Managua/16/2007 |  | Other Database Import |
| EPI160188 | NA (N2) | Nicaragua | 2007-Jun-21 | A/Managua/17/2007 |  | Other Database Import |
| EPI154036 | NA (N2) | Nicaragua | 2007-Jun-22 | A/Managua/20/2007 |  | Other Database Import |
| EPI154100 | NA (N2) | Nicaragua | 2007-Jun-23 | A/Managua/34/2007 |  | Other Database Import |
| EPI176127 | NA (N2) | Nicaragua | 2007-Jun-24 | A/Managua/1507.01/2007 |  | Other Database Import |
| EPI154446 | NA (N2) | Nicaragua | 2007-Jun-27 | A/Managua/25/2007 |  | Other Database Import |
| EPI320630 | NA (N2) | Nicaragua | 2010-Jul-02 | A/Managua/2492.04/2010 |  | Other Database Import |
| EPI274473 | NA (N2) | Nicaragua | 2010-Jun-02 | A/Nicaragua/2145/2010 | Laboratorio de Virologia, Direccion de Microbiologia | Centers for Disease Control and Prevention |
| EPI285244 | NA (N2) | Nicaragua | 2010-Jun-05 | A/Managua/3192.01/2010 |  | Other Database Import |
| EPI285468 | NA (N2) | Nicaragua | 2010-Jun-06 | A/Managua/4456.02/2010 |  | Other Database Import |
| EPI315766 | NA (N2) | Nicaragua | 2010-Jun-06 | A/Managua/5898.02/2010 |  | Other Database Import |
| EPI315774 | NA (N2) | Nicaragua | 2010-Jun-07 | A/Managua/3209.01/2010 |  | Other Database Import |
| EPI277439 | NA (N2) | Nicaragua | 2010-Jun-08 | A/Managua/5806.01/2010 |  | Other Database Import |
| EPI285260 | NA (N2) | Nicaragua | 2010-Jun-08 | A/Managua/5871.02/2010 |  | Other Database Import |
| EPI285276 | NA (N2) | Nicaragua | 2010-Jun-08 | A/Managua/194.01/2010 |  | Other Database Import |
| EPI320638 | NA (N2) | Nicaragua | 2010-Jun-30 | A/Managua/3424.01/2010 |  | Other Database Import |
| EPI320646 | NA (N2) | Nicaragua | 2011-Jan-28 | A/Managua/38-11/2011 |  | Other Database Import |
| EPI466924 | NA (N2) | Nicaragua | 2013-Jun-20 | A/Managua/1083/2013 | Laboratorio de Virologia, Direccion de Microbiologia | Centers for Disease Control and Prevention |
| EPI465946 | NA (N2) | Nicaragua | 2013-May-07 | A/Esteli/816/2013 | Laboratorio de Virologia, Direccion de Microbiologia | Centers for Disease Control and Prevention |
| EPI156038 | NA (N2) | Panama | 2007-May-22 | A/Panama/0475/2007 |  | Centers for Disease Control and Prevention |
| EPI278759 | NA (N2) | Panama | 2010-Jun-08 | A/Panama/307149/2010 | Instituto Conmemorativo Gorgas de Estudios de la Salud | Centers for Disease Control and Prevention |
| EPI467431 | NA (N2) | Panama | 2013-Jun-17 | A/Panama/313131/2013 | Instituto Conmemorativo Gorgas de Estudios de la Salud | Centers for Disease Control and Prevention |
| EPI185801 | NA (N2) | Puerto Rico | 2009-Feb-09 | A/Puerto Rico/18/2009 | Puerto Rico Department of Health | Centers for Disease Control and Prevention |
| EPI193919 | NA (N2) | Puerto Rico | 2009-Jun-02 | A/Puerto Rico/46/2009 | Puerto Rico Department of Health | Centers for Disease Control and Prevention |
| EPI193907 | NA (N2) | Puerto Rico | 2009-May-26 | A/Puerto Rico/25/2009 | Puerto Rico Department of Health | Centers for Disease Control and Prevention |
| EPI295252 | NA (N2) | Puerto Rico | 2010-Sep-17 | A/Puerto Rico/02/2010 | Puerto Rico Department of Health | Centers for Disease Control and Prevention |
| EPI346467 | NA (N2) | Puerto Rico | 2011-Aug-25 | A/Puerto Rico/03/2011 | Puerto Rico Department of Health | Centers for Disease Control and Prevention |
| EPI347535 | NA (N2) | Puerto Rico | 2011-Oct-20 | A/Puerto Rico/23/2011 | Puerto Rico Department of Health | Centers for Disease Control and Prevention |
| EPI387762 | NA (N2) | Puerto Rico | 2012-Jun-03 | A/Puerto Rico/04/2012 | Puerto Rico Department of Health | Centers for Disease Control and Prevention |
| EPI394786 | NA (N2) | Puerto Rico | 2012-Jun-14 | A/Puerto Rico/23/2012 | Puerto Rico Department of Health | Centers for Disease Control and Prevention |
| EPI394789 | NA (N2) | Puerto Rico | 2012-Jun-26 | A/Puerto Rico/34/2012 | Puerto Rico Department of Health | Centers for Disease Control and Prevention |
| EPI486427 | NA (N2) | Puerto Rico | 2013-Sep-15 | A/Puerto Rico/19/2013 | Puerto Rico Department of Health | Centers for Disease Control and Prevention |
| EPI391258 | NA (N2) | Saint Lucia | 2012-May-24 | A/St. Lucia/2073/2012 | Caribbean Epidemiology Center | Centers for Disease Control and Prevention |
| EPI185947 | NA (N2) | Trinidad and Tobago | 2009-Jan-01 | A/Trinidad/2940/2009 |  | Centers for Disease Control and Prevention |
| EPI459819 | NA (N2) | Trinidad and Tobago | 2013-Feb-28 | A/Trinidad/847/2013 | Caribbean Epidemiology Center | Centers for Disease Control and Prevention |
| EPI459822 | NA (N2) | Trinidad and Tobago | 2013-Mar-19 | A/Trinidad/1117/2013 | Caribbean Epidemiology Center | Centers for Disease Control and Prevention |
| EPI498070 | NA (N2) | Trinidad and Tobago | 2013-Oct-27 | A/Trinidad/3558/2013 | Caribbean Epidemiology Center | Centers for Disease Control and Prevention |
| EPI537014 | NA (N2) | United States | 2012-Apr-15 | A/Texas/50/2012 | Texas Department of State Health Services-Laboratory Services | Centers for Disease Control and Prevention |
