# Supplementary material for: Origin, distribution, and potential risk factors associated with influenza A virus in swine in two production systems in Guatemala
Source: Influenza Other Respir Viruses. 2017 Jan 30;11(2):182–92. doi: 10.1111/irv.12437 (PMC5304577; doi:10.1111/irv.12437)
Supplement: Supplementary file 4 [file IRV-11-182-s004.docx]

**Supplementary Material**

**Table S2.** Summary statistics of sampled pig production units, Guatemala, 2010 and 2011.

| **Year** | **Type of PPU** | **Statistics** | **Herd size** | **Sick pigs** | **Sick pigs sampled** | **Pigs sampled** |
| --- | --- | --- | --- | --- | --- | --- |
| 2010 | Backyard | Number of PPU with available data | 94 | 91 | 98 | 101 |
|  |  | Number of PPU with missing data | 7 | 10 | 3 | 0 |
|  |  | Number of pigs | 447 | 119 | 100 | 173 |
|  |  | Min | 1 | 0 | 0 | 1 |
|  |  | Max | 28 | 7 | 6 | 6 |
|  |  | Range | 27 | 7 | 6 | 5 |
|  |  | Median | 3 | 1 | 1 | 1 |
|  |  | Mean | 5 | 1 | 1 | 2 |
|  |  | Mean 95% CI | 1 | 0.4 | 0.3 | 0.2 |
|  |  | std.dev | 5 | 2 | 1 | 1 |
| 2010 | Commercial farms | |  |  |  |  |
|  |  | Number of PPU with available data | 85 | 77 | 79 | 85 |
|  |  | Number of PPU with missing data | 0 | 8 | 6 | 0 |
|  |  | Number of pigs | 9919 | 386 | 206 | 329 |
|  |  | Min | 3 | 0 | 0 | 1 |
|  |  | Max | 1800 | 32 | 10 | 10 |
|  |  | Range | 1797 | 32 | 10 | 9 |
|  |  | Median | 30 | 4 | 2 | 3 |
|  |  | Mean | 117 | 5 | 3 | 4 |
|  |  | Mean 95% CI | 57 | 1 | 1 | 0.5 |
|  |  | std.dev | 265 | 6 | 2 | 2 |
| 2011 | Backyard |  |  |  |  |  |
|  |  | Number of PPU with available data | 136 | 136 | 141 | 141 |
|  |  | Number of PPU with missing data | 5 | 5 | 0 | 0 |
|  |  | Number of pigs | 778 | 102 | 78 | 281 |
|  |  | Min | 1 | 0 | 0 | 1 |
|  |  | Max | 50 | 9 | 8 | 10 |
|  |  | Range | 49 | 9 | 8 | 9 |
|  |  | Median | 3 | 0 | 0 | 1 |
|  |  | Mean | 6 | 1 | 1 | 2 |
|  |  | Mean 95% CI | 1 | 0.3 | 0.2 | 0.3 |
|  |  | std.dev | 8 | 1 | 1 | 2 |
| 2011 | Commercial farms | |  |  |  |  |
|  |  | Number of PPU with available data | 53 | 53 | 53 | 53 |
|  |  | Number of PPU with missing data | 0 | 0 | 0 | 0 |
|  |  | Number of pigs | 5736 | 137 | 82 | 230 |
|  |  | Min | 7 | 0 | 0 | 1 |
|  |  | Max | 900 | 12 | 8 | 10 |
|  |  | Range | 893 | 12 | 8 | 9 |
|  |  | Median | 50 | 0 | 0 | 4 |
|  |  | Mean | 108 | 3 | 2 | 4 |
|  |  | Mean 95% CI | 54 | 1 | 1 | 1 |
|  |  | std.dev | 195 | 4 | 3 | 3 |

**Table S3.** Percentage of IAV positive pigs detected by rRT-PCR and their geographic distribution in Guatemala, 2010 and 2011.

| **Department** | **Backyard** | | **Farms** | | **Total** | |
| --- | --- | --- | --- | --- | --- | --- |
|  | **Positive pigs (%)** | **Sampled pigs** | **Positive pigs (%)** | **Sampled pigs** | **Positive pigs (%)** | **Sampled pigs** |
| **2010** | **51(15.5)** | **329** | **27(16)** | **169** | **78(15.7)** | **498** |
| **North** |  |  |  |  |  |  |
| Alta Verapaz | 1(8.3) | 12 | 3(25) | 12 | 4(16.7) | 24 |
| Peten | 2(13.3) | 15 | 1(8.3) | 12 | 3(11.1) | 27 |
| **Central** |  |  |  |  |  |  |
| Baja Verapaz | 3(42.9) | 7 | 1(16.7) | 6 | 4(30.8) | 13 |
| Chimaltenango | 1(3.6) | 28 | 0(-) | 3 | 1(3.2) | 31 |
| Guatemala | 3(10) | 30 | 1(12.5) | 8 | 4(10.5) | 38 |
| Sacatepequez | 0(-) | 25 | 0(-) | 4 | 0(-) | 29 |
| **East** |  |  |  |  |  |  |
| Chiquimula | 1(6.7) | 15 | 1(16.7) | 6 | 2(9.5) | 21 |
| El Progreso | 2(16.7) | 12 | 0(-) | 1 | 2(15.4) | 13 |
| Izabal | 1(12.5) | 8 | 1(50) | 2 | 2(20) | 10 |
| Jalapa | 1(11.1) | 9 | 0(-) | 1 | 1(10) | 10 |
| Jutiapa | 3(11.1) | 27 | 1(14.3) | 7 | 4(11.8) | 34 |
| Zacapa | 7(70) | 10 | 1(50) | 2 | 8(66.7) | 12 |
| **South** |  |  |  |  |  |  |
| Escuintla | 0(-) | 20 | 3(18.8) | 16 | 3(8.3) | 36 |
| Retalhuleu | 1(10) | 10 | 0(-) | 7 | 1(5.9) | 17 |
| Santa Rosa | 3(11.1) | 27 | 0(-) |  | 3(11.1) | 27 |
| Suchitepequez | 0(-) | 9 | 4(20) | 20 | 4(13.8) | 29 |
| **West** |  |  |  |  |  |  |
| Huehuetenango | 5(29.4) | 17 | 1(2.8) | 36 | 6(11.3) | 53 |
| Quetzaltenango | 2(20) | 10 | 1(16.7) | 6 | 3(18.8) | 16 |
| Quiche | 5(62.5) | 8 | 5(55.6) | 9 | 10(58.8) | 17 |
| San Marcos | 0(-) | 9 | 0(-) | 6 | 0(-) | 15 |
| Solola | 7(77.8) | 9 | 2(66.7) | 3 | 9(75) | 12 |
| Totonicapan | 3(25) | 12 | 1(50) | 2 | 4(28.6) | 14 |
| **2011** | **24(10.4)** | **230** | **33(12.8)** | **257** | **57(11.7)** | **487** |
| **North** |  |  |  |  |  |  |
| Alta Verapaz | 1(8.3) | 12 | 0(-) | 12 | 1(4.2) | 24 |
| Peten | 1(10) | 10 | 2(11.8) | 17 | 3(11.1) | 27 |
| **Central** |  |  |  |  |  |  |
| Baja Verapaz | 0(-) |  | 2(15.4) | 13 | 2(15.4) | 13 |
| Chimaltenango | 0(-) | 28 | 0(-) |  | 0(-) | 28 |
| Guatemala | 6(18.2) | 33 | 0(-) |  | 6(18.2) | 33 |
| Sacatepequez | 0(-) | 5 | 3(12.5) | 24 | 3(10.3) | 29 |
| **East** |  |  |  |  |  |  |
| Chiquimula | 0(-) | 10 | 0(-) | 11 | 0(-) | 21 |
| El Progreso | 0(-) | 10 | 0(-) | 3 | 0(-) | 13 |
| Izabal | 0(-) |  | 2(20) | 10 | 2(20) | 10 |
| Jalapa | 0(-) |  | 3(30) | 10 | 3(30) | 10 |
| Jutiapa | 1(4.2) | 24 | 0(-) | 10 | 1(2.9) | 34 |
| Zacapa | 2(33.3) | 6 | 1(25) | 4 | 3(30) | 10 |
| **South** |  |  |  |  |  |  |
| Escuintla | 4(19) | 21 | 1(6.7) | 15 | 5(13.9) | 36 |
| Retalhuleu | 0(-) | 17 | 0(-) |  | 0(-) | 17 |
| Santa Rosa | 0(-) |  | 4(14.8) | 27 | 4(14.8) | 27 |
| **West** |  |  |  |  |  |  |
| Huehuetenango | 2(14.3) | 14 | 6(15.4) | 39 | 8(15.1) | 53 |
| Quetzaltenango | 1(10) | 10 | 0(-) | 6 | 1(6.3) | 16 |
| Quiche | 2(11.8) | 17 | 0(-) |  | 2(11.8) | 17 |
| San Marcos | 4(44.4) | 9 | 0(-) | 6 | 4(26.7) | 15 |
| Solola | 0(-) | 4 | 1(12.5) | 8 | 1(8.3) | 12 |
| Suchitepequez | 0(-) |  | 5(17.9) | 28 | 5(17.9) | 28 |
| Totonicapan | 0(-) |  | 3(21.4) | 14 | 3(21.4) | 14 |

**Table S4.** Percentage of IAV positive herds detected by rRT-PCR and their geographic distribution in Guatemala, 2010 and 2011.

| **Department** | **Backyard** | | **Farms** | | **Total** | |
| --- | --- | --- | --- | --- | --- | --- |
|  | **Positive herds (%)** | **Sampled herds** | **Positive herds (%)** | **Sampled herds** | **Positive herds (%)** | **Total sampled pigs** |
| **2010** | **35(41.2)** | **85** | **22(21.8)** | **101** | **57(30.6)** | **186** |
| **North** |  |  |  |  |  |  |
| Alta Verapaz | 1(33.3) | 3 | 2(40) | 5 | 3(37.5) | 8 |
| Peten | 2(33.3) | 6 | 1(25) | 4 | 3(30) | 10 |
| **Central** |  |  |  |  |  |  |
| Baja Verapaz | 1(100) | 1 | 1(16.7) | 6 | 2(28.6) | 7 |
| Chimaltenango | 1(25) | 4 | 0(-) | 3 | 1(14.3) | 7 |
| Guatemala | 3(50) | 6 | 1(25) | 4 | 4(40) | 10 |
| Sacatepequez | 0(-) | 5 | 0(-) | 4 | 0(-) | 9 |
| **East** |  |  |  |  |  |  |
| Chiquimula | 1(16.7) | 6 | 1(16.7) | 6 | 2(16.7) | 12 |
| El Progreso | 1(50) | 2 | 0(-) | 1 | 1(33.3) | 3 |
| Izabal | 1(25) | 4 | 1(50) | 2 | 2(33.3) | 6 |
| Jalapa | 1(33.3) | 3 | 0(-) | 1 | 1(25) | 4 |
| Jutiapa | 3(100) | 3 | 1(14.3) | 7 | 4(40) | 10 |
| Zacapa | 5(100) | 5 | 1(50) | 2 | 6(85.7) | 7 |
| **South** |  |  |  |  |  |  |
| Escuintla | 0(-) | 4 | 2(50) | 4 | 2(25) | 8 |
| Retalhuleu | 1(33.3) | 3 | 0(-) | 3 | 1(16.7) | 6 |
| Santa Rosa | 2(50) | 4 | 0(-) |  | 2(50) | 4 |
| Suchitepequez | 0(-) | 3 | 3(50) | 6 | 3(33.3) | 9 |
| **West** |  |  |  |  |  |  |
| Huehuetenango | 2(50) | 4 | 1(4.8) | 21 | 3(12) | 25 |
| Quetzaltenango | 1(100) | 1 | 1(16.7) | 6 | 2(28.6) | 7 |
| Quiche | 3(100) | 3 | 4(57.1) | 7 | 7(70) | 10 |
| San Marcos | 0(-) | 4 | 0(-) | 6 | 0(-) | 10 |
| Solola | 3(100) | 3 | 1(100) | 1 | 4(100) | 4 |
| Totonicapan | 3(37.5) | 8 | 1(50) | 2 | 4(40) | 10 |
| **2011** | **17(32.1)** | **53** | **30(21.3)** | **141** | **47(24.2)** | **194** |
| **North** |  |  |  |  |  |  |
| Alta Verapaz | 1(33.3) | 3 | 0(-) | 7 | 1(10) | 10 |
| Peten | 1(25) | 4 | 2(22.2) | 9 | 3(23.1) | 13 |
| **Central** |  |  |  |  |  |  |
| Baja Verapaz | 0(-) |  | 2(40) | 5 | 2(40) | 5 |
| Chimaltenango | 0(-) | 4 | 0(-) |  | 0(-) | 4 |
| Guatemala | 4(66.7) | 6 | 0(-) |  | 4(66.7) | 6 |
| Sacatepequez | 0(-) | 1 | 3(60) | 5 | 3(50) | 6 |
| **East** |  |  |  |  |  |  |
| Chiquimula | 0(-) | 10 | 0(-) | 10 | 0(-) | 20 |
| El Progreso | 0(-) | 2 | 0(-) | 1 | 0(-) | 3 |
| Izabal | 0(-) |  | 2(66.7) | 3 | 2(66.7) | 3 |
| Jalapa | 0(-) |  | 3(30) | 10 | 3(30) | 10 |
| Jutiapa | 1(33.3) | 3 | 0(-) | 9 | 1(8.3) | 12 |
| Zacapa | 2(66.7) | 3 | 1(25) | 4 | 3(42.9) | 7 |
| **South** |  |  |  |  |  |  |
| Escuintla | 2(66.7) | 3 | 1(50) | 2 | 3(60) | 5 |
| Retalhuleu | 0(-) | 3 | 0(-) |  | 0(-) | 3 |
| Santa Rosa | 0(-) |  | 4(15.4) | 26 | 4(15.4) | 26 |
| **West** |  |  |  |  |  |  |
| Huehuetenango | 2(66.7) | 3 | 3(60) | 5 | 5(62.5) | 8 |
| Quetzaltenango | 1(100) | 1 | 0(-) | 6 | 1(14.3) | 7 |
| Quiche | 1(33.3) | 3 | 0(-) |  | 1(33.3) | 3 |
| San Marcos | 2(66.7) | 3 | 0(-) | 6 | 2(22.2) | 9 |
| Solola | 0(-) | 1 | 1(50) | 2 | 1(33.3) | 3 |
| Suchitepequez | 0(-) |  | 5(17.9) | 28 | 5(17.9) | 28 |
| Totonicapan | 0(-) |  | 3(100) | 3 | 3(100) | 3 |

**Table S5.** Mutations in swine isolates from Guatemala in comparison to co-circulating human viruses from 2009 to 2011. The mutations in bold correspond to residues that are more prevalent in swine viruses according to searches on the Influenza Research Database (www.fludb.org).

| Subtype | Gene | Mutation | Isolate ID | % in database (human strains) | % in database (swine strains) | Location |
| --- | --- | --- | --- | --- | --- | --- |
| H1N1 |  |  |  |  |  |  |
|  | PB2 | Q288R | all | 0.04 | 0.09 |  |
|  |  | Xaa (S688F) | 70167 | 0.04 | N/D |  |
|  | PA | Xaa (L589F) | 70165 | 0.02 | N/D | PB1 binding region |
|  |  | Xaa (S600F) | 70167 | 0.02 | N/D | PB1 binding region |
|  |  | **E688G** | all | 0.22 | 20.6 | PB1 binding region |
|  |  | Xaa (A689P) | 70167 | N/D | N/D | PB1 binding region |
|  | HA | **V251L** | 70166 | 0.1 | 53 | HA1 extracellular domain, beta strand |
|  |  | **R222K** | all | 3.7 | 73 | HA1 extracellular domain, beta strand. Antigenic site Ca1 |
|  | NA | **G298A** | all | 0.01 | 58.9 | Catalytic domain |
|  |  | Xaa (S340F) | 70167 | 0.46 | 0.52 | Catalytic domain |
|  |  | Xaa (**E462D**) | 70167 | 0.01 | 60.6 | Catalytic domain |
|  | M2 | T43I | all | 0.2 | 0.55 | Transmembrane domain |
|  | NS1 | V60F | all | N/D | N/D | NS1 alpha helix of RNA binding domain |
| H3N2 |  |  |  |  |  |  |
|  | PB1 | N213D | 400078 | 0.13 | 0.3 | Nuclear localization motif |
|  | PB1-F2 | 1 - 53 | 400078 | 13.39 | 0 |  |
|  | HA | A16T | 400078 | 1.11 | 0.2 | Signal peptide |
|  |  | Xaa (G234R) | 400078 | 0.16 | 0 | HA1 extracelular domain |

**Table S6**. Summary of the 14 amino acid substitutions among 040078-H3N2 and the human seasonal H3N2 vaccine strains. Amino acid residues specific of the 040078-H3N2 virus are shown in bold.

| Amino acid position | H3 numbering | A/Victoria/361/2011\|EPI101506 | A/Texas/50/2012\|EPI122006 | 040078-H3N2 | Sequence feature |
| --- | --- | --- | --- | --- | --- |
| 16 | -N/A- | A | A | **T** | Signal peptide |
| 49 | HA1: 33 | Q | R | Q |  |
| 61 | HA1: 45 | N | N | **S** |  |
| 64 | HA1: 48 | I | I | **T** |  |
| 69 | HA1: 53 | D | D | **N** | H3 beta-strand, H3 antigenic-site C |
| 110 | HA1: 94 | Y | Y | **H** |  |
| 144 | HA1: 128 | T | N | T |  |
| 214 | HA1: 198 | S | P | A | H3 beta-strand |
| 215 | HA1: 199 | S | S | **A** | H3 beta-strand |
| 234 | HA1: 218 | G | G | **R** |  |
| 239 | HA1: 223 | I | I | **V** |  |
| 246 | HA1: 230 | I | I | **V** |  |
| 294 | HA1: 278 | N | K | N | H3 beta-strand, H3 antigenic-site C |
| 296 | HA1: 280 | E | E | **A** |  |
| 328 | HA1: 312 | S | S | **N** |  |

**Figure S1.** Global Maximum Likelihood inference under the Hasegawa-Kishino-Yano (HKY) + Gamma model of nucleotide substitution of the evolutionary relationships, among A/swine/Guatemala/CIP049-040078/2010 (H3N2) (highlighted in yellow) and other swine and human viruses from 2007 to 2014. Viruses from Central America and the Caribbean are shown in color: viruses from 2007-2008 (mostly A/Brisbane/10/2007 lineage) orange, viruses from 2009 (mostly A/Perth/16/2009 lineage) green, viruses from 2010-2011 (mostly 040078-H3N2 like) red, viruses from 2012 (mostly A/Victoria/361/2011 lineage) purple, viruses from 2013-2014 (mostly A/Texas/50/2011 lineage) blue. Recent swine isolates from the USA are shown in magenta. Background viruses from other geographic locations are shown in grey. Neighbor-Joining bootstrap support values are displayed. Scale-bar depicts number of substitutions per site.

**Figure S2.** Map of Guatemala and pig density by department (number of pigs/km^2^), according to projected census data from the Guatemalan Ministry of Agriculture, Livestock and Food in 2008 (1).

**Supplementary Material References**

1. MAGA. (2013). Regional project for the prevention of classical swine fever in the republic of Guatemala [in Spanish]. 2012 Oct [cited 2016 Mar 22] http://visar.maga.gob.gt/visar/sa/ppc/proyecto-reg.pdf
